# LPF: a framework for exploring the wing color pattern formation of ladybird beetles in Python

**DOI:** 10.1093/bioinformatics/btad430

**Published:** 2023-07-08

**Authors:** Daewon Lee

**Affiliations:** School of Art and Technology, College of Art and Technology, Chung-Ang University, Anseong 17546, Republic of Korea; Graduate School of Advanced Imaging Sciences, Multimedia, and Film, Chung-Ang University, Seoul 06974, Republic of Korea

## Abstract

**Summary:**

Biological pattern formation is one of the complex system phenomena in nature, requiring theoretical analysis based on mathematical modeling and computer simulations for in-depth understanding. We propose a Python framework named LPF to systematically explore the highly diverse wing color patterns of ladybirds using reaction-diffusion models. LPF supports GPU-accelerated array computing for numerical analysis of partial differential equation models, concise visualization of ladybird morphs, and evolutionary algorithms for searching mathematical models with deep learning models for computer vision.

**Availability and implementation:**

LPF is available on GitHub at https://github.com/cxinsys/lpf.

## 1 Introduction

Biological pattern formation is one of the complex system phenomena that best demonstrates the beauty of nature (Koch and Meinhardt 1994, Meinhardt 2009). Mathematical modeling provides a framework to analyze and interpret the underlying mechanisms of biological pattern formation. The reaction–diffusion (RD) model introduced by Turing has been widely investigated to understand pattern formation, especially in developmental processes (Turing 1990, Kondo and Miura 2010). Kondo and Miura (2010) emphasize that even simple mathematical models can help reveal and explain the essential principles of real-world biological systems. For instance, the mechanism of stripe pattern formation in a tropical fish, *Pomacanthus*, was suggested by Kondo and Asai (1995), based on a two-component reaction-diffusion wave system. Sekimura *et al.* (2000) explored the wing color pattern of a butterfly, *Papilio dardanus*, through the numerical simulations of Gierer-Meinhardt model on a realistic geometry. Recently, Manukyan *et al.* (2017) have reproduced the skin color dynamics of ocellated lizards with a three-component RD model. Milinkovitch and his colleagues have further investigated the lizard skin patterns for realistic 3D geometries and multiple species of lizards (Fofonjka and Milinkovitch 2021, Jahanbakhsh and Milinkovitch 2022).

Ladybird beetles are employed as model organisms for studying intraspecific polymorphism, as the wing color patterns of ladybird species are highly diverse (Ando and Niimi 2019, Niimi and Ando 2021). Recently, Ando *et al.* (2018) have revealed *pannier* gene is the major regulator of the color pattern variation in *Harmonia axyridis*, and multiple inversions in the first intron of the gene are found in the different color morphs. This genetic role of *pannier* gene also has been confirmed by Gautier *et al.* (2018). However, there are few studies on mathematical models of ladybirds, despite being an important model organism for understanding color pattern formation in nature. Liaw *et al.* (2001) proposed a Turing model of ladybird beetles and demonstrated the patterns of some species in the 2D and spherical surface geometries. Ringham (2020) developed the 3D models of five ladybird species based on Liaw’s model.

To systematically explore the wing color patterns of ladybirds, we have developed a synthetic framework named LPF (Ladybird Pattern Formation) in Python. LPF provides numerical partial differential equation (PDE) solvers for reaction-diffusion models, which can be optimized for a batch of parameter sets by GPU computing. LPF also has visualization methods that synthesize images and videos of ladybird wing color patterns through image composition. Users can search the parameters of a mathematical model to replicate the images of target ladybirds using evolutionary algorithms with deep learning models for computer vision. We expect that various applications, from understanding the principles of pattern formation to studying artificial life, will be supported by LPF framework (Ando and Niimi 2019, Gershenson *et al.* 2020).

## 2 Results

### 2.1 Basic concepts

A PDE model is a mathematical model used to describe how a function changes over space and time. Generally, biological pattern formation can be modeled by PDEs. Since most RD models contain nonlinear terms, we usually obtain approximate solutions using numerical methods. In order to generate the wing color patterns of ladybirds, we can first consider the RD model. Liaw *et al.* (2001) developed a Turing RD model derived from Gierer–Meinhardt model and demonstrated that the model could generate various patterns of different ladybird beetles (Gierer and Meinhardt 1972, Liaw *et al.* 2001). Liaw model is a two-component PDE defined by Equation (1).


(1) $$\begin{array}{c} \frac{\partial u}{\partial t}=D_{u}\nabla^{2}u+\rho_{u}\frac{u^{2}v}{1+\kappa u^{2}}+\sigma_{u}-\mu u,\\ \frac{\partial v}{\partial t}=D_{v}\nabla^{2}v-\rho_{v}\frac{u^{2}v}{1+\kappa u^{2}}+\sigma_{v}, \end{array}$$

where *u* and *v* are the state variables, and $D_{u}$  $D_{v}$ are diffusion parameters. The other parameters in Equation (1), $\rho_{u}$, $\rho_{v}$, $\sigma_{u}$, $\sigma_{v}$, κ, and μ, are the kinetic parameters of the reactions. To efficiently explore the wing color patterns of ladybirds with a PDE model like Equation (1), users can utilize representative RD models in 2D space rather than incorporating complicated systems and 3D realistic geometries. LPF is designed to be object-oriented, where users can easily extend various types of objects for dynamics models, initial conditions, numerical solvers, and optimization methods in Python. LPF also provides wrapper objects for array computing to apply hardware-driven optimizations such as GPU acceleration.

### 2.2 Workflow

The primary workflow of the LPF framework involves conducting a simulation experiment and subsequently visualizing the results (Supplementary Figs S1–S5). The initial step is configuring a simulation experiment, which includes defining spatiotemporal parameters with selecting a suitable computing device. The next step is creating an initializer and an array of parameter sets. The initializer knows how to initialize the states in the 2D space. Based on the initializer and the parameter array, a PDE model object is created, which can describe the RD dynamics of the wing color pattern (Supplementary Table S1). Finally, we can perform a simulation experiment using numerical methods for solving differential equations (Supplementary Table S2). Visualizing the numerical simulation results makes it easier to obtain critical interpretations and insights.

### 2.3 GPU acceleration

Solving the Liaw model in a 128 × 128 spatial domain using the Euler method in LPF on a single core of a 3.4 GHz CPU typically requires 2–3 min for 500 000 iterations for a single parameter set. Consequently, generating results for 100 parameter sets requires about 3–5 h of computational time. To efficiently explore the various color patterns of ladybirds, we adopt parallel processing of multiple parameter sets based on GPU computing. Performance tests demonstrate that we can achieve at most 77× performance improvement with GPU acceleration compared to a single CPU core (Supplementary Figs S6 and S7).

### 2.4 Visualization

**Figure 1.**
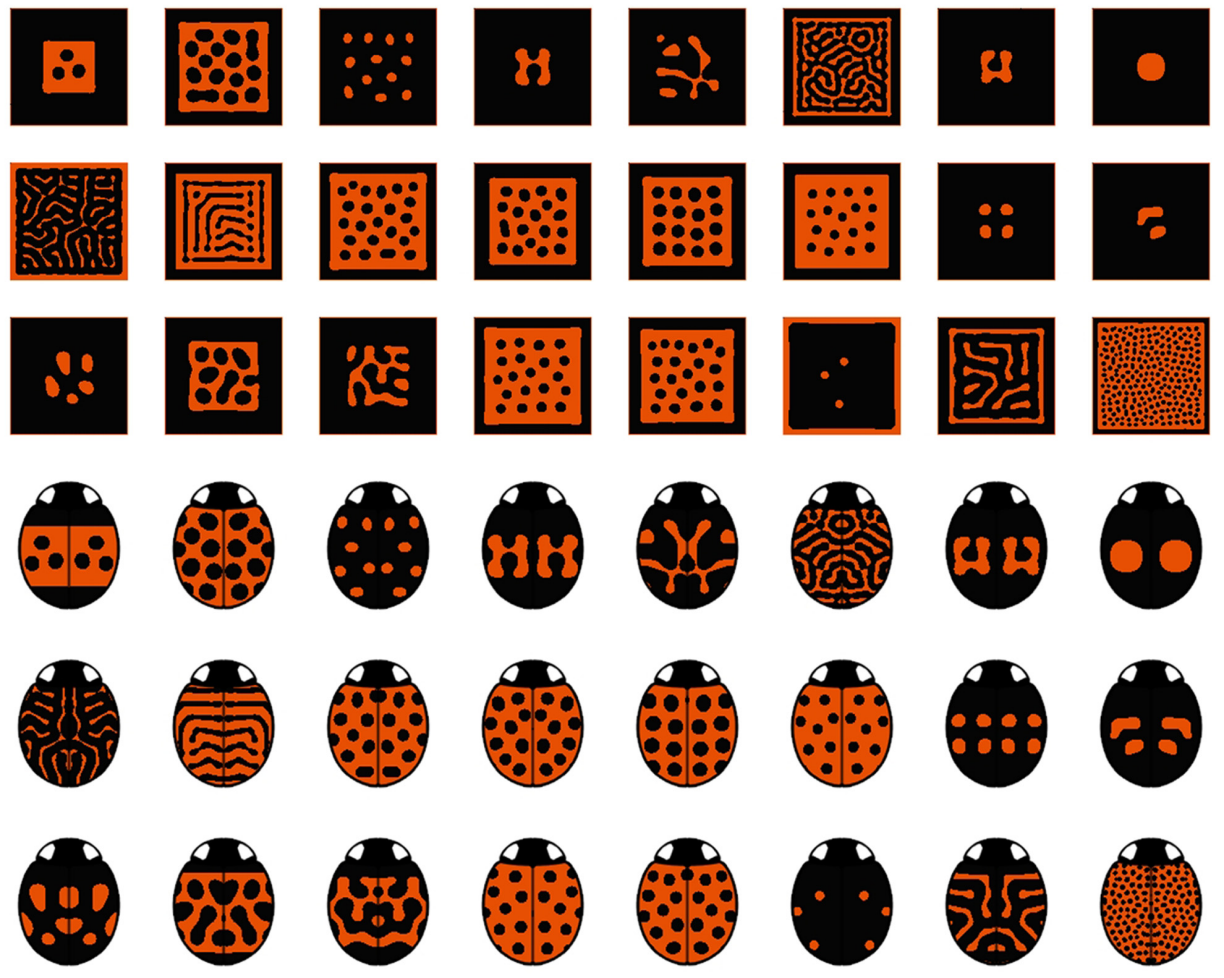
The examples of patterns (top) and ladybird morphs (bottom) for *Harmonia axyridis* generated based on Liaw’s model in the LPF framework (Liaw *et al.*, 2001).

A concise and cartoon-style visualization for model organisms can provide more insights than visualizing 2D square images of arbitrary colors or 3D-rendered images with too much detail. LPF visualizes both patterns and ladybird morphs through image composition (Fig. 1 and Supplementary Figs S8–S13). The ladybird image templates of LPF have been designed to reflect the way experimental biologists represent ladybirds in conceptual diagrams (Gautier *et al.* 2018, Ando and Niimi 2019). Figure 1 shows only red and black color patterns that are originated from melanin and carotenoid pigments of *H.axyridis*, respectively, but users can change the color maps for their target ladybirds in LPF.

### 2.5 Evolutionary search

Finding initial conditions and parameter values of a PDE model that can replicate the patterns of target organisms requires extensive computer simulations. LPF provides evolutionary search methods based on PyGMO for discovering the initial conditions and parameters (Biscani and Izzo 2020). Users can also adopt perceptual losses or learned perceptual image patch similarity (LPIPS) based on deep learning models for computer vision (Johnson *et al.* 2016, Zhang *et al.* 2018). These deep learning models help define objective functions that measure how much synthetic images are close to target images (Supplementary Figs S14–S21, Tables S3 and S4).

### 2.6 Diploid model

To reflect the genetic features of *H.axyridis* in inheritance (Tan and Li 1934, Tan 1946, Komai 1956, Ando *et al.* 2018, Gautier *et al.* 2018, Ando and Niimi 2019), we have developed diploid models (Supplementary Fig. S22). In the crossing experiments, we observe that a diploid model without crosstalks better reproduces Tan’s ‘mosaic dominance’ phenomena (Supplementary Fig. S23). To implement the evolution of a population, we devise an *in silico* crossover, where paternal and maternal haploid models undergo crossover in a diploid model (Supplementary Fig. S24). The experiments of population evolution demonstrate the mosaic dominance in the early generations and a kind of genetic drift in the late generations (Supplementary Figs S25 and S26).

## 3 Conclusion

LPF is a framework that provides functionalities to explore the highly diverse wing color patterns of ladybirds. To efficiently search the patterns in LPF, we have focused on simple RD systems and concise visualization. LPF will support the studies for adopting mathematical models to analyze intraspecific polymorphism using ladybirds as model organisms.

## Supplementary Material

btad430_Supplementary_Data

## Data Availability

LPF is available at https://github.com/cxinsys/lpf.
