# Supplementary material for: LPF: a framework for exploring the wing color pattern formation of ladybird beetles in Python
Source: Bioinformatics. 2023 Jul 8;39(7):btad430. doi: 10.1093/bioinformatics/btad430 (PMC10354001; doi:10.1093/bioinformatics/btad430)
Supplement: btad430_Supplementary_Data [file btad430_supplementary_data.pdf]

Supplementary Data

LPF: a framework for exploring the wing color pattern formation of ladybird beetles in Python

Daewon Lee[1,2]

[1] *School of Art and Technology, College of Art and Technology, Chung-Ang University, Anseong, Republic of Korea*

[2] *Graduate School of Advanced Imaging Sciences, Multimedia, and Film, Chung-Ang University, Seoul, Republic of Korea*

Contents

1 Introduction **2**

2 Numerical simulation **3**

2.1 Basic workflow 3

2.1.1 Configuring a simulation experiment 6

2.1.2 Creating an initializer 10

2.1.3 Creating an array of parameter sets 10

2.1.4 Creating a PDE model 12

2.1.5 Performing a numerical simulation 13

2.1.6 Visualizing the results 15

2.2 GPU acceleration for a batch of parameter sets 18

3 Pattern visualization **21**

3.1 Visualizing a single morph 21

3.2 Visualizing multiple morphs 24

3.3 Creating a video for temporal evolution 24

4 Evolutionary search **28**
4.1 Parameter optimization 28
4.2 Fitness score 33
4.3 Results of a case study: reproducing schematic images 34
4.4 Results of a case study: reproducing real images 43

5 Diploid model **45**
5.1 Numerical simulation 46
5.2 Population evolution 52

1 Introduction

We provide a GitHub repository to facilitate the open-source project of LPF (Ladybird Pattern Formation) framework. You can find the implementation in Python and helpful tutorials to get started with the LPF framework in the repository.

- Home: `https://github.com/cxinsys/lpf`
- Tutorials: `https://github.com/cxinsys/lpf/tree/main/tutorials`

In this supplementary data, we introduce LPF framework, which is developed to explore the wing color pattern formation of ladybird beetles using Python. LPF framework has the following main features.

1. Reaction-diffusion PDE models and numerical methods.
2. GPU acceleration of the PDE solvers for a batch of parameter sets.
3. Visualization of the wing color patterns of various morphs.
4. Evolutionary search for discovering the parameter sets of a PDE model.
5. Diploid models for analyzing population evolution in crossing experiments.

2 Numerical simulation

2.1 Basic workflow

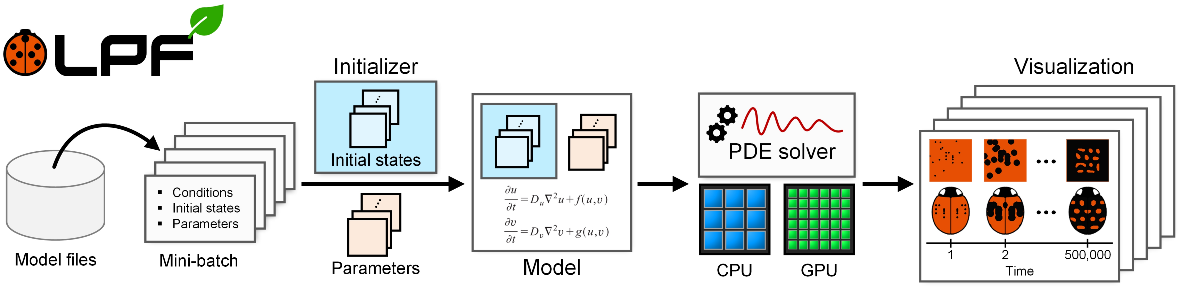

Figure S1. A workflow of simulation experiment in LPF.

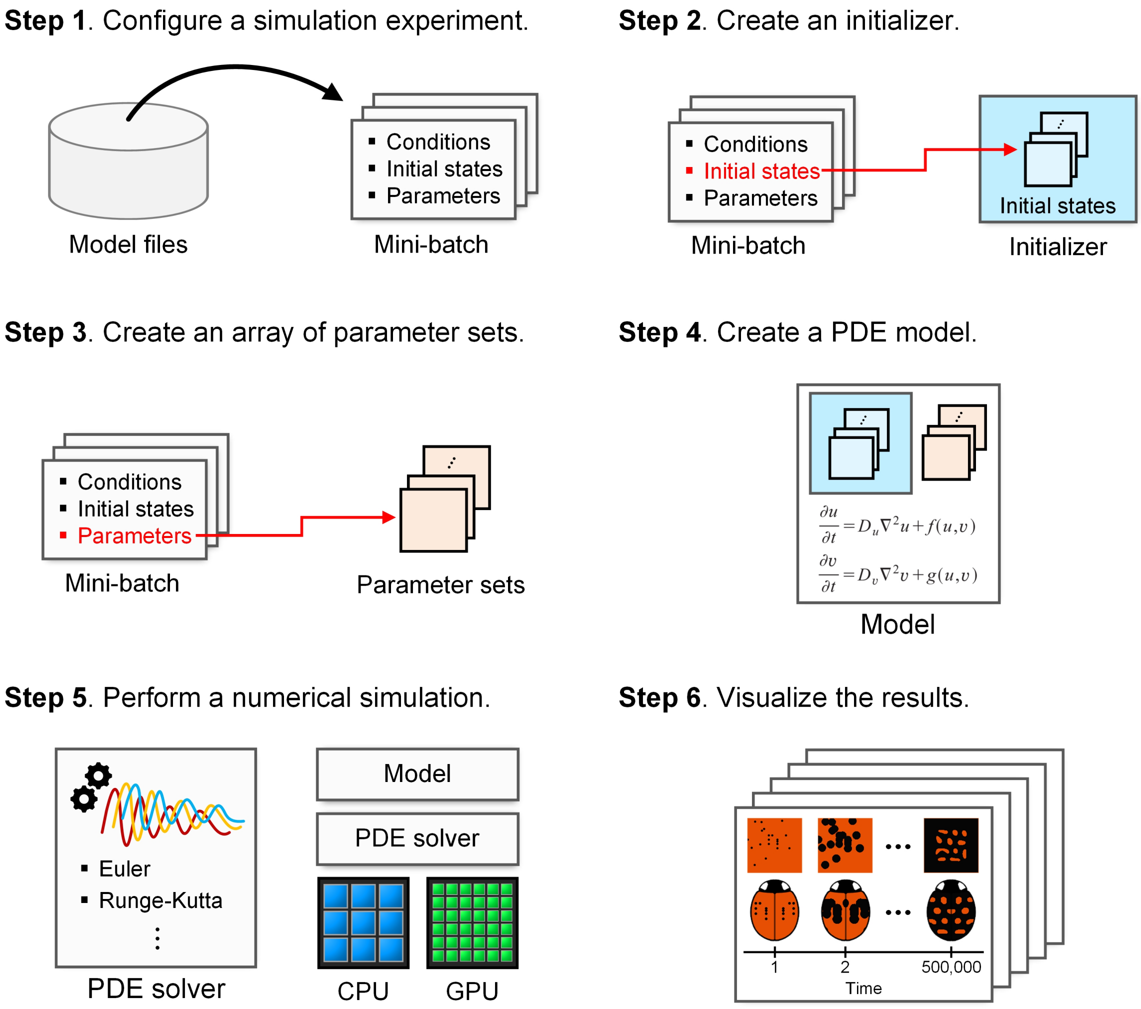

Figure S2. The steps of simulation experiment workflow in LPF.

Code 1. How to obtain a numerical solution for a single set of parameters.

```python
import os
import os.path as osp
import time
from datetime import datetime

import numpy as np

from lpf.initializers import LiawInitializer
from lpf.models import LiawModel
from lpf.solvers import EulerSolver
from lpf.visualization import merge_single_timeseries

# Computing device (CPU or GPU)
device = "cpu"

# Time parameters
dt = 0.01
n_iters = 500000

# Space parameters
dx = 0.1
width = 128
height = 128
shape = (height, width)

# Create the output directory.
str_now = datetime.now().strftime('%Y%m%d-%H%M%S')
dpath_output = osp.join("./output", "result_%s" % (str_now))
os.makedirs(osp.abspath(dpath_output), exist_ok=True)

# Create a dict for parameters.
param_dict = {
    "u0": 2.0, "v0": 1.0,
    "Du": 0.0005, "Dv": 0.075,
    "ru": 0.18, "rv": 0.02874,
    "su": 0.001, "sv": 0.025,
    "k": 0.084,
    "mu": 0.08
}

# In this example, we use random positions for initializing u with u0.
for i in range(25):
    param_dict["init_pts_%d"%(i+1)] = (np.random.randint(0, height),
                                       np.random.randint(0, width))

```

```python
model_dicts = []
model_dicts.append(param_dict)

# Create the Liaw initializer.
initializer = LiawInitializer()
initializer.update(model_dicts)

# Create an array of parameter sets.
params = LiawModel.parse_params(model_dicts)

# Create the Liaw model.
model = LiawModel(
    initializer=initializer,
    params=params,
    dx=dx,
    width=width,
    height=height,
    device=device
)

# Create the Euler solver.
solver = EulerSolver()

# Get the numerical solution.
solver.solve(
    model=model,
    dt=dt,
    n_iters=n_iters,
    period_output=1000,
    dpath_model=dpath_output,
    dpath_ladybird=dpath_output,
    dpath_pattern=dpath_output,
    verbose=1
)

# Visualize the temporal evolution by merging images.
dpath_images = pjoin(dpath_output, "model_1")

img_patterns = merge_single_timeseries(dpath_input=dpath_images,
                                       n_cols=10,
                                       infile_header="pattern",
                                       ratio_resize=0.5,
                                       text_format="n = ",
                                       font_size=10,
                                       text_margin_ratio=.1)

img_patterns.save(pjoin(dpath_output, "output_pattern.png"))

```

```
img_ladybirds = merge_single_timeseries(dpath_input=dpath_images,
                                        n_cols=10,
                                        infile_header="ladybird",
                                        ratio_resize=0.5,
                                        text_format="n = ",
                                        font_size=10,
                                        text_margin_ratio=.1)

img_ladybirds.save(pjoin(dpath_output, "output_ladybird.png"))
```

2.1.1 Configuring a simulation experiment

First, users should set parameters related to time and space after importing essential packages (Code 2). `dt` and `n_iters` represent the time step size and the number of iterations in the numerical solution. The appropriate value for the time step, `dt`, will depend on the problem being solved and the desired level of accuracy. The larger the time step, the faster the simulation will run, but the less accurate it will be. The smaller the time step, the more accurate the simulation, but it will also take longer. It should be noted that too large `dt` has the potential to give rise to numerical inaccuracies and errors, which may ultimately compromise the the results of numerical simulations.

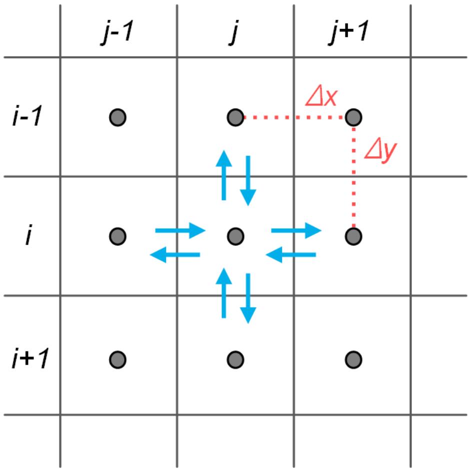

Figure S3. A 2D space of the reaction-diffusion system. The 2D space is discretized by a square grid. Blue arrows represent diffusion, and red lines represent space step size.

The Laplace operator ∇^2 for 2D Cartesian space is defined by Eq. (1). The second partial derivatives of Laplace operator can be approximated by the second-order centered difference in a 2D square grid (Figure S3, Eqs. (2) and (3)). Δx and Δy are the

space step sizes in the 2D discrete space, representing the distance between each spatial element on the x and y axes.

$$\nabla^2 u = \frac{\partial^2 u}{\partial x^2} + \frac{\partial^2 u}{\partial y^2}. \tag{1}$$

$$\left(\frac{\partial^2 u}{\partial x^2}\right)_{i,j} \approx \frac{u_{i,j-1} - 2u_{i,j} + u_{i,j+1}}{\Delta x^2}, \tag{2}$$

$$\left(\frac{\partial^2 u}{\partial y^2}\right)_{i,j} \approx \frac{u_{i-1,j} - 2u_{i,j} + u_{i+1,j}}{\Delta y^2}. \tag{3}$$

$$\begin{aligned} \nabla^2 u &\approx \frac{u_{i,j-1} - 2u_{i,j} + u_{i,j+1}}{\Delta x^2} + \frac{u_{i-1,j} - 2u_{i,j} + u_{i+1,j}}{\Delta y^2}, \\ &= \frac{u_{i,j-1} + u_{i,j+1} + u_{i-1,j} + u_{i+1,j} - 4u_{i,j}}{\Delta x^2}, \end{aligned} \tag{4}$$

where Δx and Δy are equal. The size of 2D space is determined by `width` and `height`, which correspond to the x and y axes in Code 2. Like the time step size, the space step size will affect the accuracy and computational time. The smaller the space step size, the more elements where the solution is calculated, resulting in a more accurate solution but also a longer computational time. Conversely, the larger the space step size, the fewer elements where the solution is calculated, resulting in a faster computation time but a less accurate solution. We define the space of the Liaw model as a square shape in LPF, so `dx` determines both space step sizes of the 2D Cartesian space (i.e., $\Delta x = \Delta y$ in Eqs. (2) and (3)).

The output directory is created based on the current timestamp, which would help distinguish the results of different simulation experiments. However, users can define the output directory path according to their own intentions. We can use `os.makedirs` function, which creates a new directory at the given path when it does not exist.

The initial concentrations of u and v and the kinetic parameters of the reactions can be defined using a Python dictionary for readability. `param_dict` is a dictionary that holds parameters for a specific model, such as initial values, diffusion constants, and initializing positions. In this example, we define the parameters of the Liaw model that incorporates `u0`, `v0` (initial values), `Du`, `Dv` (diffusion constants), `ru`, `rv`, `su`, `sv`, `k`, and `mu` (kinetic parameters). We also generate 25 random positions for initializing the 2D space of `u` with `u0`. Note that we append a single parameter set, `param_dict` to a list named `model_dicts`. The size of the list `model_dicts` determines the batch size.

Users also can utilize predefined model settings by loading model files. The file format of model files in LPF is JSON (JavaScript Object Notation), which is one of the most popular data interchange formats. Code 3 shows an example of Liaw model setting defined in JSON format. LPF also provides a utility function to load multiple model files to create `model_dicts` (Code 4).

Code 2. Configuring a simulation experiment.

```
import os
import os.path as osp
import time
from datetime import datetime

import numpy as np

# Computing device (CPU or GPU)
device = "cpu"

# Time parameters
dt = 0.01
n_iters = 500000

# Space parameters
dx = 0.1
width = 128
height = 128
shape = (height, width)

# Create the output directory.
str_now = datetime.now().strftime('%Y%m%d-%H%M%S')
dpath_output = osp.join("./output", "result_%s" % (str_now))
os.makedirs(osp.abspath(dpath_output), exist_ok=True)

# Create a dict for parameters.
param_dict = {
    "u0": 2.0, "v0": 1.0,
    "Du": 0.0005, "Dv": 0.075,
    "ru": 0.18, "rv": 0.02874,
    "su": 0.001, "sv": 0.025,
    "k": 0.084,
    "mu": 0.08
}

# In this example, we use random positions for initializing u with u0.
for i in range(25):
    param_dict["init_pts_%d"%(i+1)] = (np.random.randint(0, height),
                                       np.random.randint(0, width))
```

```
model_dicts = []
model_dicts.append(param_dict)
```

Code 3. An example of Liaw model setting defined in JSON format.

```
{
    "width": 128,
    "height": 128,
    "dx": 0.1,
    "dt": 0.01,
    "n_iters": 500000,
    "color_u":
    "color_v":
    "thr": 0.5,
    "initializer": "LiawInitializer",
    "u0": 2.0,
    "v0": 1.0,
    "Du": 0.0005,
    "Dv": 0.075,
    "ru": 0.18,
    "rv": 0.029,
    "k": 0.2,
    "su": 0.001,
    "sv": 0.025,
    "mu": 0.08,
    "init_pts_0": ["40", "34"],
    "init_pts_1": ["20", "58"],
    "init_pts_2": ["47", "50"]
}
```

Code 4. Loading model files.

```
import os
from lpf.data import load_model_dicts

LPF_REPO_HOME = ... # Specify the directory path of LPF repository.
dpath_model_files = os.path.join(LPF_REPO_HOME
                                 "population",
                                 "init_pop_succinea")

# Create model_dicts by loading model files.
model_dicts = load_model_dicts()
```

2.1.2 Creating an initializer

The initializers take the parameters and prepare them for use in the simulation. LPF provides initializer classes, which define initializing rules. For example, `LiawInitializer` initializes u with u_0 only for user-defined positions, while it assigns the same value v_0 to all points of v in the 2D space. However, `TwoComponentConstantInitializer` assigns u_0 and v_0 to all points of u and v in the 2D space, respectively (Figure S4). The initializer is then updated with `param_dict` using the `update` method. Users can define their custom initializer class by deriving `lpf.initializer.Initializer` and defining `update`, `initialize`, and `to_dict` methods.

Code 5. Creating an initializer.

```python
from lpf.initializers import LiawInitializer

initializer = LiawInitializer()
initializer.update(model_dicts)
params = LiawModel.parse_params(model_dicts)
```

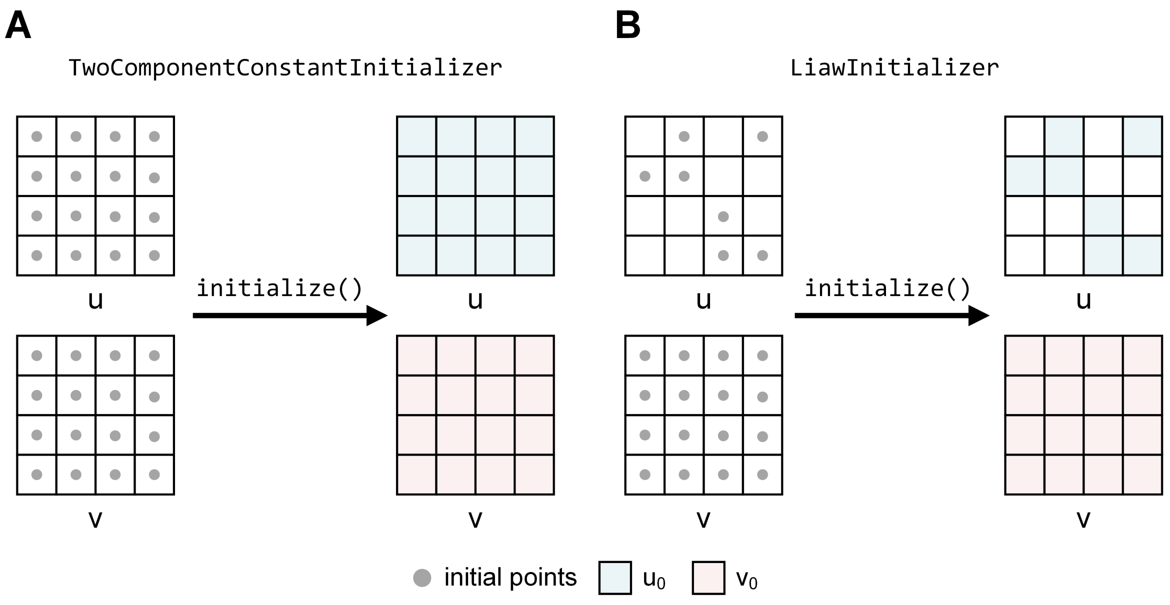

Figure S4. Initialization of a two-component 2D space by initializer objects in LPF such as **(A)** `TwoComponentConstantInitializer` and **(B)** `LiawInitializer`.

2.1.3 Creating an array of parameter sets

The diffusion and kinetic parameters defined in a dictionary should be converted to an array in which each parameter has a predefined index. In LPF, model classes can parse

the dictionary that contains their parameter values. In Code 6, `LiawModel` class parses the values of parameters, and we create an array of `numpy`, named `params` from the returned value. Note that the model class, not the model instance, parses parameter values using `parse_params` static method. We can create our array without parsing the dictionary if we assign the parameter values to the exact positions in the array. It is important to note that the `params` variable in Code 6 does not hold information about the initial state and initializing points. Code 7 is the definition of `parse_params` method of `LiawModel`, which shows a model class how to parse its diffusion and kinetic parameters from the given model dictionaries to create an `numpy.ndarray` object.

Code 6. Creating an array of parameter sets by parsing the model dictionaries.

```
from lpf.models import LiawModel

params = LiawModel.parse_params(model_dicts)
```

Code 7. The definition of `parse_params` method of `LiawModel`.

```
import numpy as np
from lpf.models import TwoComponentModel

class LiawModel(TwoComponentModel):

    @staticmethod
    def parse_params(model_dicts):
        """Parse the parameters from the model dictionaries.
           A model knows how to parse its parameters.
        """
        batch_size = len(model_dicts)
        params = np.zeros((batch_size, 8), dtype=np.float64)

        for index, n2v in enumerate(model_dicts):
            params[index, 0] = n2v["Du"]
            params[index, 1] = n2v["Dv"]
            params[index, 2] = n2v["ru"]
            params[index, 3] = n2v["rv"]
            params[index, 4] = n2v["k"]
            params[index, 5] = n2v["su"]
            params[index, 6] = n2v["sv"]
            params[index, 7] = n2v["mu"]

        return params
```

2.1.4 Creating a PDE model

Table S1 shows examples of PDE models in the LPF framework, such as Gierer-Meinhardt [Gierer and Meinhardt, 1972], Gray-Scott [Gray and Scott, 1984], Liaw [Liaw et al., 2001], and Schnakenberg [Schnakenberg, 1979] models. Users can choose from the models defined in LPF, as well as define their own models. To define a custom model class derived from `TwoComponentModel`, we should define four methods: `__init__`, `reactions`, `to_dict`, and `parse_params` (Code 8). If we want to perform the evolutionary search of LPF with our custom model, we have to define two additional methods, `get_param_bounds` and `len_decision_vector`. The most important one among the above methods is `reactions` method, as it defines our equations.

Code 10 demonstrates how to create a texttttLiawModel object. The model constructor receives `initializer`, `params`, and space parameters to instantiate the model. Note that we define the computing device (CPU or GPU) as a parameter of the model constructor. If users have multiple parameter sets, it is much more efficient to use the GPU based on CuPy [Okuta et al., 2017].

Table S1. PDE models defined in LPF.

Model	Class	Reactions	Parameters
Gierer-Meinhardt	`GiererMeinhardtModel`	$f(u,v) = \rho_u \frac{u^2}{v} - \mu u$<br>$g(u,v) = \rho_v u^2 - \nu v$	ρ_u: `ru`, ρ_v: `rv`,<br>μ: `mu`, ν: `nu`
Gray-Scott	`GrayScottModel`	$f(u,v) = -u^2 v + F(1-u)$<br>$g(u,v) = u^2 v - (F+k)v$	F: `F`, k: `k`
Liaw	`LiawModel`	$f(u,v) = \rho_u \frac{u^2 v}{1+\kappa u^2} + \sigma_u - \mu u$<br>$g(u,v) = -\rho_v \frac{u^2 v}{1+\kappa u^2} + \sigma_v$	ρ_u: `ru`, ρ_v: `rv`, κ: `k`,<br>σ_u: `su`, σ_v: `sv`, μ: `mu`
Schnakenberg	`SchnakenbergModel`	$f(u,v) = \sigma_u - \mu u + \rho u^2 v$<br>$g(u,v) = \sigma_v - \rho u^2 v$	σ_v: `sv`, σ_u: `su`,<br>ρ: `rho`, μ: `mu`

Code 8. The outline of a custom PDE model class.

```python
from lpf.models import TwoComponentModel

class MyModel(TwoComponentModel):

    def __init__(self, *args, **kwargs):
        super().__init__(*args, **kwargs)
        self._name = "MyModel"

    def reactions(self, t, u_c, v_c):
        """Define the reactions of a two-component system.
        """
```

```python
        return f, g

    def to_dict(self, *args, **kwargs):
        """Create a dict to store parameter values.
        """
        return n2v

    @staticmethod
    def parse_params(model_dicts):
        """Parse the parameter sets from model dictionaries.
        """
        return params

    def get_param_bounds(self):
        """The bounds of the decision vector in EvoSearch.
        """
        return bounds_min, bounds_max

    def len_decision_vector(self):
        """The length of the decision vector in EvoSearch.
        """
        return 0
```

Code 9. An example of creating a Liaw model.

```python
from lpf.models import LiawModel

# Create the Liaw model.
model = LiawModel(
    initializer=initializer,
    params=params,
    dx=dx,
    width=width,
    height=height,
    device=device
)
```

2.1.5 Performing a numerical simulation

The numerical solvers perform numerical simulations for a given model with time parameters. We can utilize numerical solvers such as Euler and Runge-Kutta methods in LPF (Table S2). In Code 10, we use Euler solver as an example. Users need to instantiate the solver object and call `solve` method of the solver object to perform the simulation experiment. The solver receives directory paths, which are the output

directories containing the 2D pattern images (`dpath_pattern`), ladybird morph images (`dpath_ladybird`), and model information files (`dpath_model`). The `verbose` parameter controls the level of standard outputs during the simulation experiment. Note that we do not define boundary conditions, which are crucial for the final results. For simplicity, LPF does not allow user-defined boundary conditions and uses Neumann boundary conditions for all models. Eq. (5) is the Neumann boundary conditions of two-component model.

$$\begin{aligned} \text{boundary}\left(\frac{\partial u}{\partial t}\right) &= 0 \quad \rightarrow \quad u'(0:h-1,\ 0:w-1) = 0, \\ \text{boundary}\left(\frac{\partial v}{\partial t}\right) &= 0 \quad \rightarrow \quad v'(0:h-1,\ 0:w-1) = 0, \end{aligned} \tag{5}$$

where w and h represent width and height of the 2D space.

Table S2. Numerical methods implemented in LPF.

Method	Class	Definition
Euler	`EulerSolver`	$y_{n+1} = y_n + h \cdot f(t, y_n)$
Heun	`HeunSolver`	$k_1 = h \cdot f(t, y_n)$<br>$k_2 = h \cdot f(t+h, y_n + k_1)$<br>$y_{n+1} = y_n + \frac{k_1+k_2}{2}$
Runge-Kutta	`RungeKuttaSolver`	$k_1 = h \cdot f(t, y_n)$<br>$k_2 = h \cdot f(t+\frac{h}{2}, y_n + \frac{k_1}{2})$<br>$k_3 = h \cdot f(t+\frac{h}{2}, y_n + \frac{k_2}{2})$<br>$k_4 = h \cdot f(t+h, y_n + k_3)$<br>$y_{n+1} = y_n + \frac{k_1+2k_2+2k_3+k_4}{6}$

Code 10. Performing a numerical simulation.

```python
from lpf.solvers import EulerSolver

# Create a solver and perform a numerical simulation.
solver = EulerSolver()

solver.solve(
    model=model,
    dt=dt,
    n_iters=n_iters,
    period_output=1000,
    dpath_model=dpath_output,
    dpath_ladybird=dpath_output,
    dpath_pattern=dpath_output,
    verbose=1
)
```

2.1.6 Visualizing the results

Code 11 shows how to visualize the numerical solution results created by merging the output images. Specifically, we create the two images for the temporal evolution of the pattern and ladybird morph. The output directory structure of simulation results for a single parameter set is as follows.

```
<OUTPUT_DIR>
├── model_1
│   ├── ladybird_000001.png
│   ├── pattern_000001.png
│   ├── ladybird_000002.png
│   ├── pattern_000002.png
│   ├── ladybird_000003.png
│   ├── pattern_000003.png
│   └── ⋮
└── models
    └── model_1.json
```

`merge_single_timeseries` function finds image files that start with a string defined by `infile_header` in the specified output directory `dpath_input` and merges them into a single image object (i.e., `PIL.Image.Image`). We can also save the `PIL.Image.Image` object using `save` method as PNG image file, and Figure S5 shows the example images. Refer to 3 Pattern visualization for more details of the visualization mechanism.

Code 11. Visualizing the temporal evolution of pattern and ladybird morph.

```
from os.path import join as pjoin
from lpf.visualization import merge_single_timeseries

dpath_images = pjoin(dpath_output, "model_1")

img_patterns = merge_single_timeseries(dpath_input=dpath_images,
                                       n_cols=10,
                                       infile_header="pattern",
                                       ratio_resize=0.5,
                                       text_format="n = ",
                                       font_size=10,
                                       text_margin_ratio=0.1)

img_patterns.save(pjoin(dpath_output, "output_pattern.png"))

img_ladybirds = merge_single_timeseries(dpath_input=dpath_images,
                                        n_cols=10,
                                        infile_header="ladybird",
                                        ratio_resize=0.5,
                                        text_format="n = ",
                                        font_size=10,
                                        text_margin_ratio=0.1)

img_ladybirds.save(pjoin(dpath_output, "output_ladybird.png"))
```

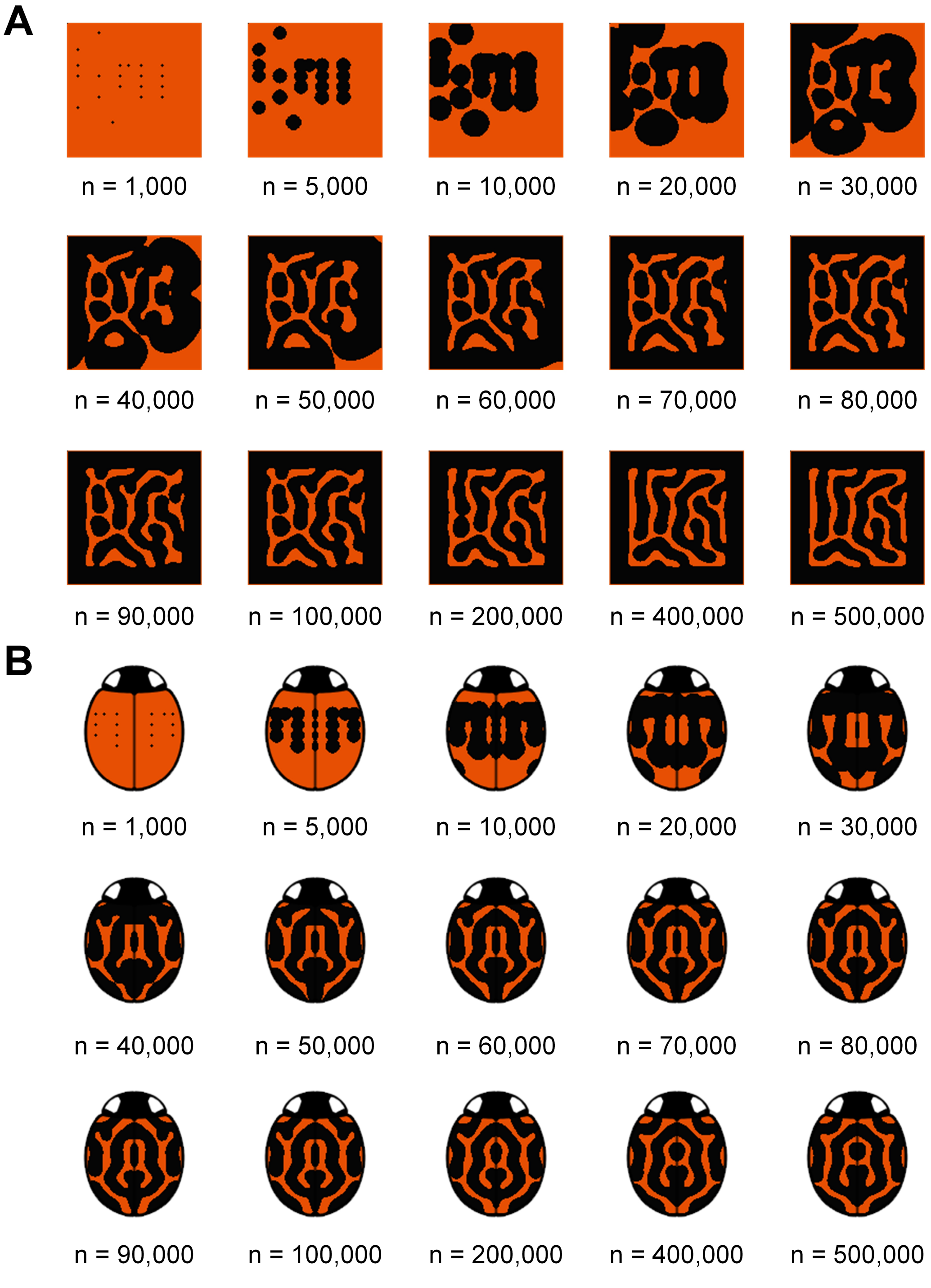

Figure S5. A visualization of the temporal evolution of **(A)** color pattern and **(B)** ladybird morph obtained from a simulation experiment of Code 1. n denotes the n-th iteration in the numerical simulation.

2.2 GPU acceleration for a batch of parameter sets

Solving the Liaw model in 128×128 space on a single core of 3.4 GHz CPU typically takes about 2-3 minutes for 500,000 iterations to obtain the result of a single parameter set. Therefore, it takes about 3-5 hours to obtain the results of 100 parameter sets on the single core of the CPU. Since we pursue rapidly exploring a variety of phenotypes rather than obtaining highly sophisticated and accurate results for a single parameter set, we adopted parallel processing of a batch of parameter sets based on GPU acceleration in the LPF framework. Recently, there have been many open-source Python projects supporting GPU acceleration for array computing. For example, we can perform parallel processing using CuPy in LPF to utilize NVIDIA CUDA-enabled GPUs [Okuta et al., 2017]. The axis of parallelization in LPF is the first axis of a batch of parameter sets. In other words, LPF parallelizes the numerical simulations for a batch of parameter sets through GPU computing. Code 12 is an example of getting the numerical solution for a batch of parameter sets.

Code 12. An example of solving the Liaw model for a batch of parameter sets.

```
import numpy as np

from lpf.initializers import LiawInitializer
from lpf.models import LiawModel
from lpf.solvers import EulerSolver

device = "cuda:0"  # Device option: CPU or GPU; we use GPU in this example.

# Time parameters
dt = 0.01
n_iters = 500000

# Space parameters
dx = 0.1
width = 128
height = 128
shape = (height, width)

# Create the Liaw initializer.
initializer = LiawInitializer()

# Load the previously defined parameters of a population.
LPF_REPO_HOME = ... # Specify the directory path of LPF repository.
dpath_init_pop = f"{LPF_REPO_HOME}/population/init_pop_axyridis/"
model_dicts = load_model_dicts(dpath_init_pop)

```

```python
# Update the initializer and parse the parameter sets.
initializer.update(model_dicts)
params = LiawModel.parse_params(model_dicts)

# Create the Liaw model.
model = LiawModel(
    initializer=initializer,
    params=params,
    dx=dx,
    width=width,
    height=height,
    device=device
)

# Create the Euler solver.
solver = EulerSolver()

# Get the numerical solution.
solver.solve(model=model,
             dt=dt,
             n_iters=n_iters,
             period_output=1000,
             verbose=1)
```

We conducted numerical simulations for 100 iterations increasing the batch size to understand performance improvement by GPU computing. We repeated each simulation ten times and measured the average and standard deviation of runtimes. The other conditions are the same as those of Code 1. The following is main hardware and software information in this experiment.

- CPU: Intel® Core™ i9-12900KS (3.4GHz)
- GPU: NVIDIA GeForce RTX™ 3090 Ti
- RAM: 4 × Samsung DDR5-4800 32GB (4000Mhz)
- OS: Windows 10 (64bits)
- SW: Python 3.9.15; NumPy 1.23.3; CuPy 10.6.0

When we ignore file input/output ('file I/O') during simulation, GPU computing improves the performance of numerical simulation for batch sizes greater than 1 (Figure S6 and Figure S7A). Note that the maximum relative performance is 77.34× achieved by the Euler method under the condition of batch size = 16 and no file I/O. Although the Runge-Kutta method basically takes a longer time than the Euler method, the performance improvement is similar to the Euler method (Figure S6 and Figure S7A).

However, GPU acceleration does not improve performance if we export pattern and ladybird images every iteration (Figure S6 and Figure S7B). Therefore, file I/O should be avoided as long as possible if we want to improve the performance of numerical simulation.

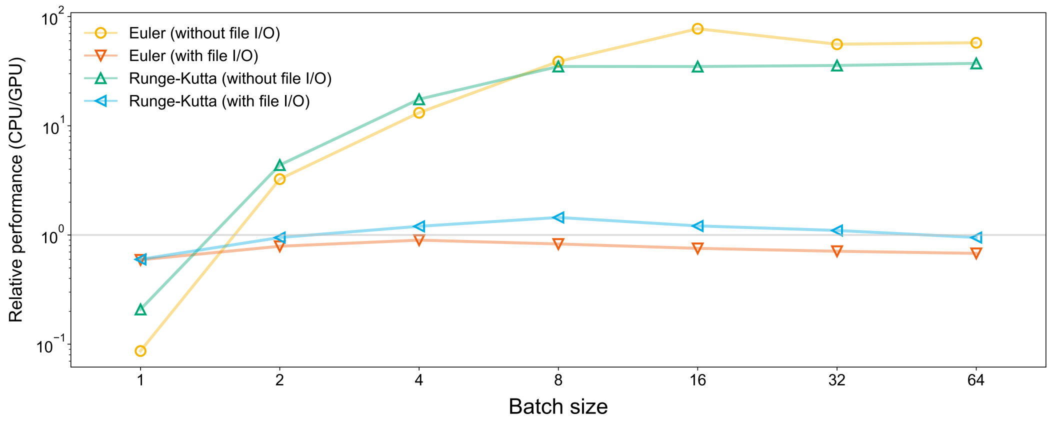

Figure S6. Relative performance of GPU compared to CPU.

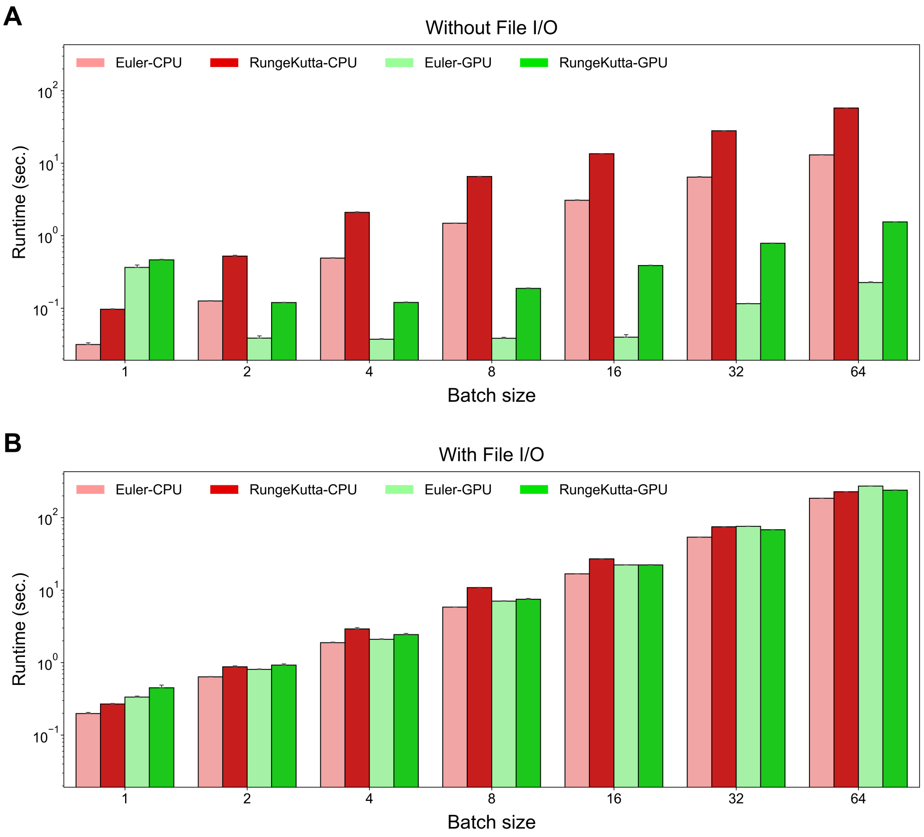

Figure S7. Runtimes of numerical solvers for different batch sizes. 'File I/O' represents saving the images of ladybird morphs and patterns at each time step in numerical analysis.

3 Pattern visualization

Although there are many ways to visualize the morphology of ladybirds, we focused only on the elytra (i.e., forewings) of ladybirds where color pattern polymorphism is prominent (Figure S8). Inspired by the phenotype pictures of Gautier *et al.* [Gautier et al., 2018], we have devised a novel method to create simplified images that display the color patterns of ladybird elytra.

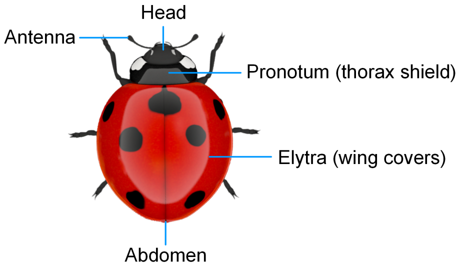

Figure S8. A concise anatomy of ladybird.

3.1 Visualizing a single morph

Interestingly, the wing color patterns of ladybirds are almost symmetric, like a decalcomania [Ando et al., 2018]. Therefore, in this study, we also assumed that the wing color patterns of ladybirds are symmetric based on the evidence from images and literature. Ocklenburg and Mundorf suggest that symmetry and modularity in biological systems arise not only from natural selection but also from their less specific information encoding, leading to a higher probability of symmetry [Ocklenburg and Mundorf, 2022]. Extensive biological data, including protein complexes, RNA secondary structures, and a model gene regulatory network, support this hypothesis, showing a distinct bias towards phenotypes with simpler and more symmetric structures, suggesting that lower descriptive complexity might favour the evolution of complex modular assemblies. Although Ocklenburg and Mundorf did not directly analyze the color patterns of insects, we propose that their hypothesis can be applied to the wing color patterns of ladybirds as gene regulatory networks regulate the phenotypes.

Visualizing a single morph of a ladybird in LPF consists of several steps of image processing after solving a PDE model. First, we colorize the 2D space according to the key states after solving a model. For example, we assume that state u is the key state that determines melanin synthesis in the Liaw model (Figure S9). We can assign a black color representing melanin to certain positions of state u greater than a threshold. Second, we crop the predefined region from the colorized pattern image to extract the wing color pattern. Third, we composite the cropped wing pattern with a ladybird

template image to generate half of the ladybird wing pattern image. Finally, we merge the left and right halves of ladybird wing patterns to create a complete image of the ladybird morph. Code 13 explains how to visualize a single morph after solving a model in LPF, and Figure S10 is an example output of Code 13. Figure S11 shows a workflow of visualizing a ladybird morph.

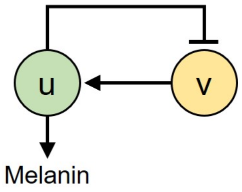

Figure S9. Melanin synthesis regulated by u in a two-component model.

Code 13. Creating the ladybird and pattern images after solving a model.

```
# A model knows how to colorize its states.
arr_color = model.colorize(thr_color=0.5)

# Create the PIL image objects from the RGBA numpy.ndarray.
img_ladybird, img_pattern = model.create_image(index=0, arr_color=arr_color)

# We can also save the ladybird and pattern images to files.
model.save_image(index=0,
                 fpath_ladybird=pjoin(dpath_output, "ladybird.png"),
                 fpath_pattern=pjoin(dpath_output, "pattern.png"),
                 arr_color=arr_color)
```

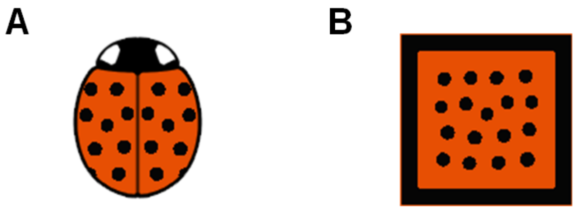

Figure S10. An example of **(A)** morph and **(B)** pattern images created in Code 13.

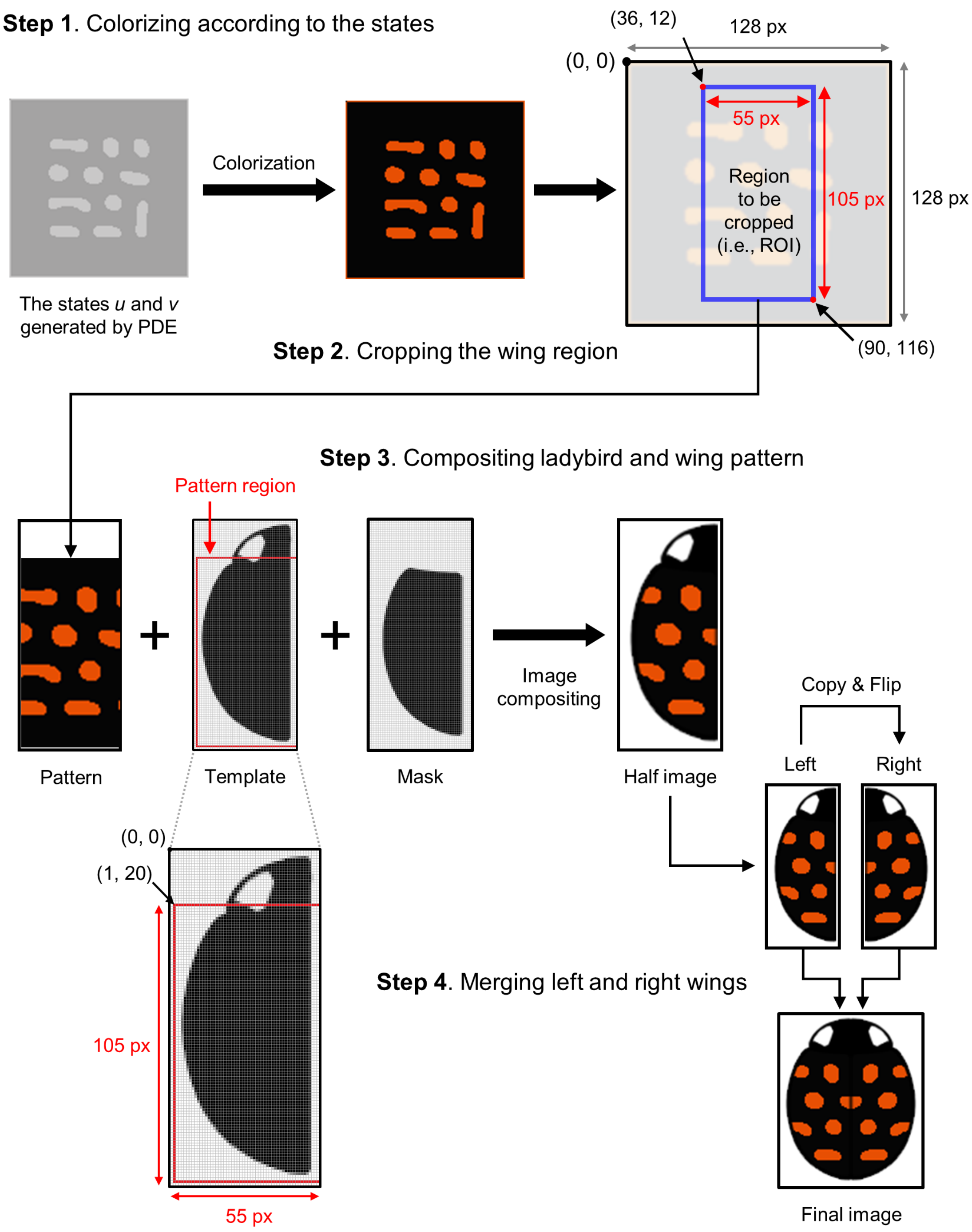

Figure S11. A workflow of visualizing a ladybird morph.

3.2 Visualizing multiple morphs

Visualizing the multiple morphs is one of the most frequently used features in the LPF framework when exploring the wing color pattern polymorphism of a ladybird species. Code 14 is an example of visualizing the multiple morphs after solving the Liaw model for a batch of parameter sets in Code 12, and Figure S12 is the result image. Note that we can also add an identifier text to `merge_multiple` function.

Code 14. Visualizing the multiple morphs of the Liaw model.

```python
# A model knows how to colorize its states.
arr_color = model.colorize(thr_color=0.5)

imgs = []
for i in range(arr_ladybird.shape[0]):
    img_ladybird, img_pattern = model.create_image(i, arr_ladybird)
    imgs.append(img_ladybird)

img_output = merge_multiple(imgs=imgs,
                            n_cols=4,
                            ratio_resize=2.0,
                            text_format="morph = ",
                            font_size=32)
# Save the image file.
img_output.save("morphs.png", dpi=(600, 600))
```

3.3 Creating a video for temporal evolution

We can see the following directory tree in the output directory after solving a model for a batch of 8 parameter sets.

```
<OUTPUT_DIR>
├── model_1
├── model_2
├── model_3
├── model_4
├── model_5
├── model_6
├── model_7
├── model_8
└── models
```

The `models` directory contains model JSON files that include metadata, parameter values, and initializing positions. `model_*` directory contains the ladybird images of each

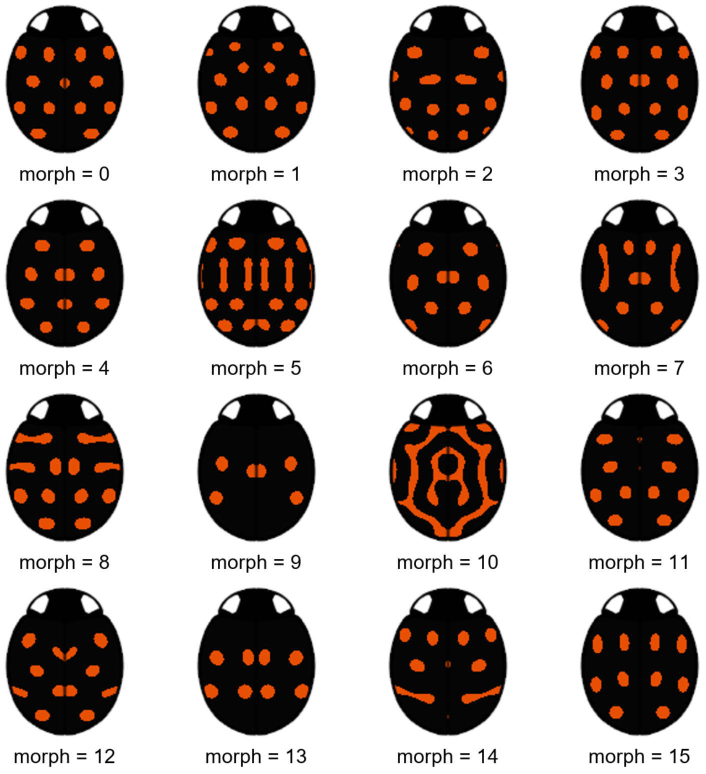

Figure S12. An example of visualizing multiple morphs of the Liaw model.

model generated during the numerical simulation. Code 15 demonstrates how to create a video that visualizes the temporal evolution of multiple morphs. `merge_multiple_timeseries` function concatenates multiple morph images into a frame and performs the concatenation over time. Technically, `merge_multiple_timeseries` function merges the multiple image files, which have the same file name in different `model_*` directories, into a single image. `create_video` function merges the frames and creates a video depending on MoviePy [Zulko, 2020]. For example, we create a MP4 video for 8 ladybirds in Code 15.

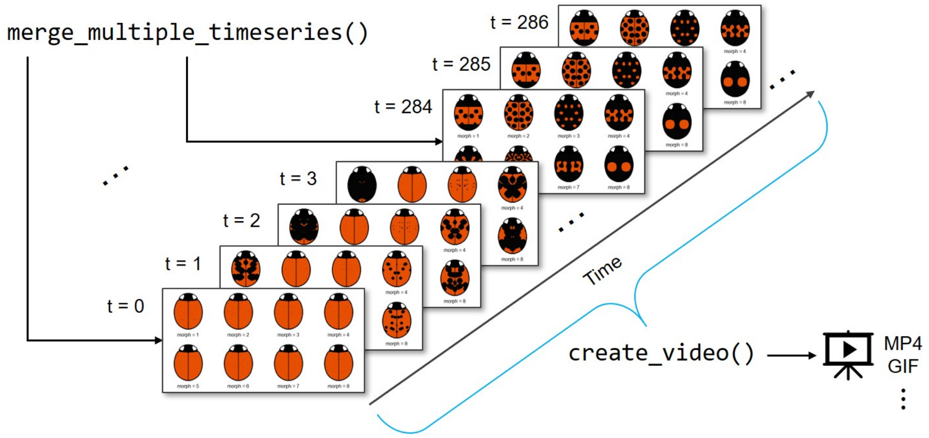

Figure S13. How `merge_multiple_timeseries` and `create_video` functions work to create a video for the temporal evolution of multiple morphs.

Code 15. Creating a video for the evolution of multiple morphs.

```
import os
import os.path as osp
from os.path import join as pjoin
from datetime import datetime

import numpy as np

from lpf.data import load_model_dicts
from lpf.initializers import LiawInitializer
from lpf.models import LiawModel
from lpf.solvers import EulerSolver
from lpf.visualization import merge_multiple_timeseries
from lpf.visualization import create_video

# Select device: CPU or GPU.
device = "cuda:0"

# Time parameters
dt = 0.01
n_iters = 500000

# Space parameters
dx = 0.1
width = 128
height = 128
shape = (height, width)

# Create the output directory.
str_now = datetime.now().strftime('%Y%m%d-%H%M%S')
dpath_output = pjoin(osp.abspath("./output"),
                     "experiment_batch_%s" % (str_now))
os.makedirs(dpath_output, exist_ok=True)

# Load a population of previously defined models.
LPF_REPO_HOME = ... # Specify the directory path of LPF repository.
dpath_pop = pjoin(LPF_REPO_HOME, "population", "test_pop_01")
model_dicts = load_model_dicts(dpath_pop)

# Create the Liaw initializer.
initializer = LiawInitializer()
initializer.update(model_dicts)
params = LiawModel.parse_params(model_dicts)

# Create the Liaw model.
model = LiawModel(initializer=initializer,
```

```
                     params=params,
                     width=width,
                     height=height,
                     dx=dx,
                     device=device)

# Create the Euler solver and solve the model.
solver = EulerSolver()
solver.solve(model=model,
             dt=dt,
             n_iters=n_iters,
             period_output=100,
             dpath_ladybird=dpath_output)

# Merge the multiple morphs into a frame.
dpath_frames = osp.join(dpath_output, "frames")
imgs = merge_multiple_timeseries(dpath_input=dpath_output,
                                 dpath_output=dpath_frames,
                                 n_cols=4,
                                 ratio_resize=1.0,
                                 text_format="morph = ",
                                 font_size=16)

# Create a video merging the frames.
create_video(dpath_frames, "video_morphs.mp4", fps=32, duration=0.1)
```

4 Evolutionary search

Users can explore a space of parameters for a mathematical model to replicate the target ladybirds. LPF provides evolutionary search algorithms implemented based on PyGMO, which is the Python wrapper of PaGMO [Biscani and Izzo, 2020]. We can also utilize deep learning models of computer vision to measure the similarity between synthetic and target images in the evolutionary search. Figure S14 shows a workflow of searching the parameters of a model for *H. axyridis*.

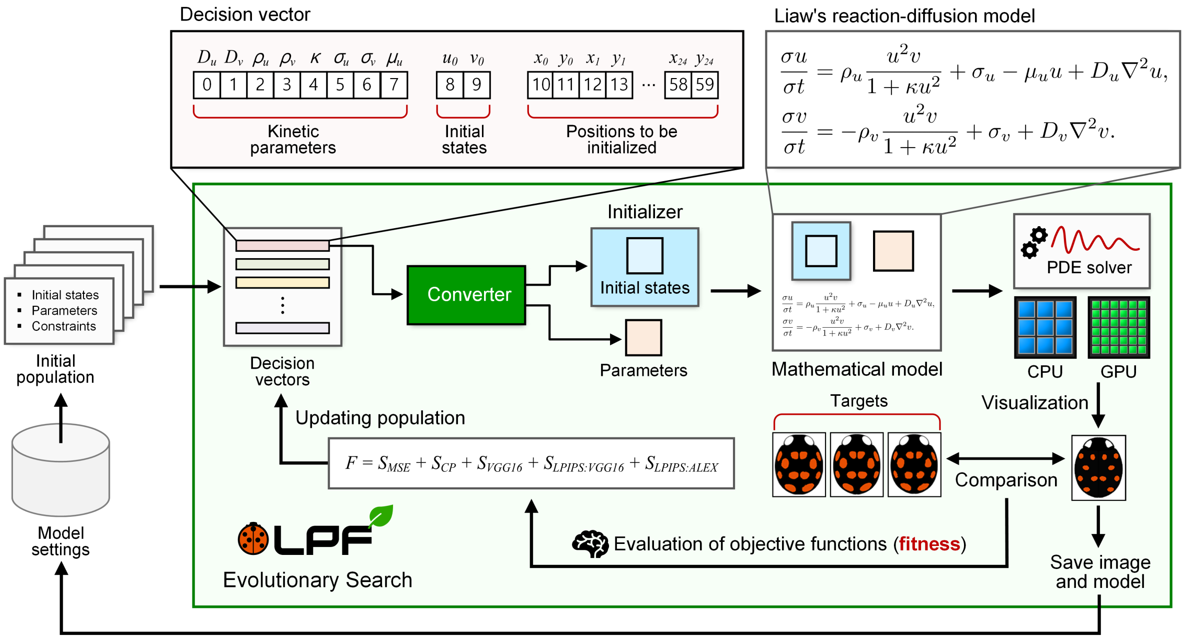

Figure S14. A workflow of searching mathematical models for *H. axyridis*.

4.1 Parameter optimization

We define the following steps to optimize the parameter set of a model using the evolutionary search algorithm of LPF.

- User selects an evolutionary algorithm in PyGMO and creates the related objects such as population, algorithm, and island.
- User initializes the population using predefined models.
- User creates a converter object in LPF that knows how to map the decision vector of the search algorithm to the parameters of a mathematical model and vice versa.
- Search algorithm solves the PDE model for a parameter set generated by the search algorithm.

- Search algorithm creates a synthetic morph image and measures the similarity between the synthetic and target images.
- Search algorithm interprets the similarity metric as a fitness score.
- Search algorithm updates the population.
- User determines whether to continue the evolution process.

Code 16. Searching the parameters of a model for replicating targets.

```
import os
import os.path as osp
from os.path import abspath as apath
import time

import numpy as np
import pygmo as pg

from lpf.data import load_model_dicts
from lpf.data import load_targets
from lpf.models import ModelFactory
from lpf.solvers import SolverFactory
from lpf.converters import ConverterFactory
from lpf.objectives import ObjectiveFactory
from lpf.search import EvoSearch

# Set the directory path of LPF repository.
LPF_REPO_HOME = osp.abspath("..")

# Create a model.
dx = 0.1
width = 128
height = 128
n_init_pts = 25

model = ModelFactory.create(
    name="Liaw",
    n_init_pts=n_init_pts,
    width=width,
    height=height,
    dx=dx
)

# Create a solver.
dt = 0.01
n_iters = 500000
```

```python
solver = SolverFactory.create(name="Euler", dt=dt, n_iters=n_iters)

# Create a converter.
converter = ConverterFactory.create("LiawInitializer")

# Create objectives.
obj_config = [
    ['MeanMeanSquareError', '1e-1', 'cpu'],
    ['MeanColorProportion', '1e0', 'cpu'],
    ['MeanVgg16PerceptualLoss', '1e-4', 'cuda:0'],
    ['MeanLearnedPerceptualImagePatchSimilarity:vgg', '1.5e1', 'cuda:0'],
    ['MeanLearnedPerceptualImagePatchSimilarity:alex', '4e0', 'cuda:0']
]

objectives = ObjectiveFactory.create(obj_config)

# Load the target laybirds.
targets = load_targets("haxyridis", ["axyridis"])

# Create an evolutionary search problem.
droot_output = osp.join(LPF_REPO_HOME, "search", "output")

search = EvoSearch(model=model,
                   solver=solver,
                   converter=converter,
                   targets=targets,
                   objectives=objectives,
                   droot_output=droot_output)

prob = pg.problem(search)

# Create the initial population.
t_beg = time.time()
pop_size = 16  # Population size
pop = pg.population(prob, size=pop_size)
dvs = []

# Initialize the population with axyridis subtype.
for i, param_dict in enumerate(model_dicts):
    if i >= pop_size:
        break
    dv = converter.to_dv(param_dict, n_init_pts)
    print(f"[DECISION VECTOR #{i+1}]\n", dv)
    pop.set_x(i, dv)
# end of for
t_end = time.time()

print("[POPULATION INITIALIZATION COMPLETED]")
```

```
print("- DURATION OF INITIALIZING POPULATION: %.3f sec."%(t_end - t_beg))
print(pop)

# Create an evolutionary algorithm.
n_procs = 4
n_gen = 100

udi = pg.mp_island()
udi.resize_pool(n_procs)

algo = pg.algorithm(pg.sade(gen=1))
isl = pg.island(algo=algo, pop=pop, udi=udi)
print(isl)

# Start searching.
try:
    for i in range(n_gen):
        t_beg = time.time()
        isl.evolve()
        isl.wait_check()
        t_end = time.time()

        print("[EVOLUTION #%d] Best objective: %f (%.3f sec.)"\
              %(i + 1, pop.champion_f[0], t_end - t_beg))

        # Save the best.
        pop = isl.get_population()
        search.save("best",
                    pop.champion_x,
                    generation=i+1,
                    fitness=pop.champion_f[0])

        # Save the population.
        arr_x = pop.get_x()
        arr_f = pop.get_f()
        for j in range(arr_x.shape[0]):
            x = arr_x[j]
            fitness = arr_f[j, 0]
            search.save("pop", x, generation=i+1, fitness=fitness)
    # end of for
except Exception as err:
    print(err)
    udi.shutdown_pool()
    raise err

print("[EVOLUTIONARY SEARCH COMPLETED]")
udi.shutdown_pool()
```

Code 17. Fitness function EvoSearch class in LPF.

```
class EvoSearch:

    def fitness(self, x):
        digest = get_hash_digest(x)

        if digest in self.cache:
            arr_color = self.cache[digest]
        else:
            x = x[None, :]
            initializer = self.converter.to_initializer(x)
            params = self.converter.to_params(x)

            self.model.initializer = initializer
            self.model.params = params

            try:
                self.solver.solve(self.model)
            except (ValueError, FloatingPointError) as err:
                print("[ERROR IN FITNESS EVALUATION]", err)
                return [np.inf]

            # Colorize the ladybird model.
            arr_color = self.model.colorize()

            # Store the colorized object in the cache.
            self.cache[digest] = arr_color
        # end of if-else

        # Evaluate objectives.
        ladybird, pattern = self.model.create_image(0, arr_color)
        sum_obj = 0
        for obj in self.objectives:
            val = obj.compute(ladybird.convert("RGB"), self.targets)
            sum_obj += val

        return [sum_obj]
```

Code 16 is an example code of the evolutionary search implementing the above steps, and Code 17 is a fitness function. We can select the evolutionary algorithm of PyGMO. In Code 16, we use a self-adaptive differential evolution (`pygmo.sade`). However, we can also replace it with a particle swarm optimization (`pygmo.pso`). We can create `pygmo.problem` object with any object that defines a function named `fitness`. The `fitness` function receives a decision vector from the search algorithm and evaluates the

fitness or objective score of the decision vector. `Converter` object knows how to map the decision vector, an array of floating-point numbers, to model parameters and vice versa. For example, `LiawConverter.to_params()` maps the first and second values in the decision vector to the diffusion parameters of `LiawModel`, D_u and D_v, respectively (Code 17). `LiawConverter.to_initializer()` instantiates `LiawInitializer` from the decision vector (Code 17).

4.2 Fitness score

The `EvoSearch` object uses single or multiple objectives to evaluate the fitness score of a decision vector. We can define a fitness score as Eq. (6).

$$F = c_1 \cdot S_{\text{MSE}} + c_2 \cdot S_{\text{CP}} + c_3 \cdot S_{\text{VGG16}} + c_4 \cdot S_{\text{LPIPS:VGG16}} + c_5 \cdot S_{\text{LPIPS:ALEX}}, \tag{6}$$

where S represents a similarity score, and c is a coefficient. S_{MSE} is a similarity score based on the mean squared error (MSE), which is defined as the mean of MSEs for multiple target images.

$$S_{\text{MSE}} = \frac{1}{m} \sum_{i=1}^{m} \text{MSE}(X, T(i)), \tag{7}$$

where m is the number of targets. The MSE between morph and target images is defined as follows.

$$\text{MSE}(X, T) = \frac{1}{n} \sum_{j=1}^{n} \left(X_j - T_j\right)^2, \tag{8}$$

where X and T are morph and target images, and n is the number of pixels in an image (e.g., $n = 128 \times 128 \times 3$).

S_{CP} is a similarity score based on color proportion. Color proportion in LPF is implemented by calculating the percentage of pixels in a particular color range out of all the pixels in an image. We use `inRange` function of OpenCV to calculate the percentage [Bradski, 2000]. S_{CP} can be defined using the inverse of normal distribution so that smaller S_{CP} represents higher similarity.

$$pdf(x, \mu, \sigma) = \frac{1}{\sigma\sqrt{2\pi}} e^{-\frac{1}{2}\left(\frac{x-\mu}{\sigma}\right)^2}, \tag{9}$$

$$S_{\text{CP}} = \frac{1}{m} \sum_{i=1}^{m} \frac{1}{pdf(\text{CP}(X), \text{CP}(T(i)), 0.1)}, \tag{10}$$

where pdf is the probability density function of normal distribution, and CP is color

proportion.

S_{VGG16} is a similarity score based on VGG16 perceptual loss [Ledig et al., 2017]. Specifically, we define the VGG16 perceptual loss as the least absolute errors (LAE) or L1 loss between the feature maps of morph and target images.

$$S_{\mathrm{VGG16}} = \frac{1}{m} \sum_{i=1}^{m} \sum_{j=1}^{4} \text{L1-loss}\left(\phi_j(X) - \phi_j(T(i))\right), \tag{11}$$

where ϕ_j is the j-th block of feature maps extracted from the VGG16 network layers.

S_{LPIPS} is a similarity score based on learned perceptual image patch similarity (LPIPS), which is similar to S_{VGG16} [Zhang et al., 2018]. LPIPS also utilizes the feature maps of a deep learning model to compute the perceptual similarity. Zhang *et al.* demonstrated that LPIPS of neural networks including SqueezeNet (Squeeze–and–excitation network, SE), AlexNet, and VGG outperformed previous metrics on large datasets.

We have investigated various metrics to compute similarity between morph and target images. To create the target images of *H. axyridis*, we have drawn three cartoon images for each subtype of *H. axyridis* We tried to reflect the characteristics of the wing color patterns, which are actually rendered in the 3D morphology of ladybirds in nature, into the 2D flattened image. (Figure S15), inspired by Gautier *et al.* [Gautier et al., 2018]. Table S3 and Table S4 show similarity scores between eight morphs and targets computed using seven metrics. All metrics give a reasonable estimate that morph-1 with two small spots is the most similar to conspicua among all targets. However, when the two red spots are relatively large, CP could not exactly compute the similarity score, as it predicts morph-2 is the most similar to axyridis, which has 12 red spots. In the case of morph-3, which is the most similar to spectabilis with four red spots, MSE, CP, SSIM, and VGG16PL fail to capture the similarity. However, LPIPS metrics with three convolutional neural networks accurately describe the similarity. Compared to morph-3, the spacing between the four red spots in morph-4 is somewhat narrower. Thus all metrics but LPIPS:ALEX cannot distinguish the number of spots, while LPIPS:ALEX suggests morph-4 is the most similar to spectabilis. The results of morph-5 and morph-6 are similar to those of morph-3, but in this case, VGG16PL also correctly predicts the similarity scores of morph-5 and morph-6. All metrics except MSE predict that morph-7 and morph-8 are the most similar to succinea. These results suggest that the MSE is not sufficient to measure the similarity of the spopt patterns of ladybirds.

4.3 Results of a case study: reproducing schematic images

We have examined succinea subtype of *H. axyridis* based on the evolutionary search. As we can see in Table S3 and Table S4, the metrics have different orders of magnitude,

so we can adjust the orders of magnitude and importance of the metrics by setting the appropriate coefficients in Eq. (6). For example, we can define the fitness for evolutionary search as follows.

$$F = 1 \times 10^{-1} \cdot S_{\mathrm{MSE}} + 1 \cdot S_{\mathrm{CP}} + 1 \times 10^{-4} \cdot S_{\mathrm{VGG16}} + 1.5 \times 10^{1} \cdot S_{\mathrm{LPIPS:VGG16}} + 4 \cdot S_{\mathrm{LPIPS:ALEX}}. \qquad (12)$$

We performed an evolutionary search for the four subtypes of *H. axyridis*, using the Liaw model and the fitness score defined by Eq. (12). Figure S16 and Figure S17 show the search results for the mathematical models of axyridis and succinea, respectively. In the first generation, some morphs were omitted because the parameter sets generated by the decision vector caused critical numerical errors. We can see that the population has reached the maximum size 16, and whole black morphs have gradually disappeared over the generations for both axyridis and succinea (Figure S16 and S17). In the 200th generation for axyridis and the 500th generation for succinea, we can observe morphs similar to the targets, although they do not perfectly match the patterns of the targets. We also performed an evolutionary search for conspicua and spectabilis of *H. axyridis*. Additionally, we conducted an evolutionary exploration for the conspicua and spectabilis subtypes of *H. axyridis* (Figure S18 and S19). For these two subtypes, we initialized the initial population with 16 morphs derived from the search for axyridis. Therefore, there is no omitted morph in the first generation of both subtypes, as the parameter sets of the initial population have been previously evaluated. Since both conspicua and spectabilis subtypes have relatively simpler patterns, we can see that the population is filled with morphs similar to the targets in the 800th generation. These results suggest that we can find the parameter sets of mathematical models that can generate the wing color patterns of ladybirds similar to target images based on the evolutionary search with the fitness score evaluated by deep learning models in LPF.

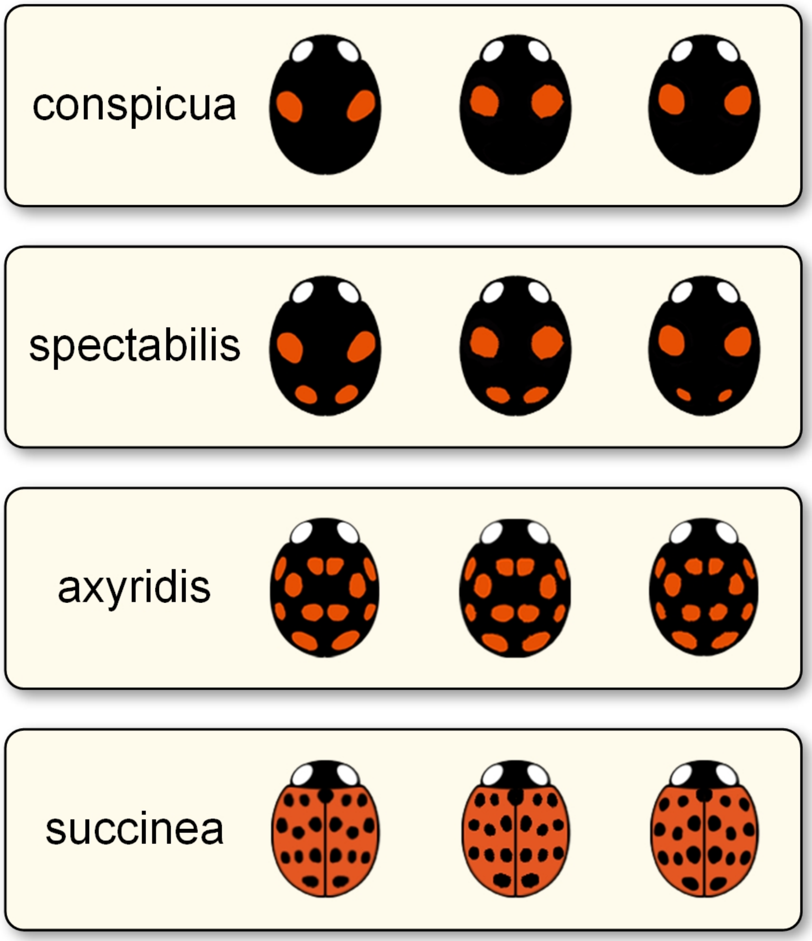

Figure S15. The schematic target images of *H. axyridis* used in evolutionary search examples. We have drawn three cartoon images for each subtype of *H. axyridis*, inspired by Gautier *et al.*, "The Genomic Basis of Color Pattern Polymorphism in the Harlequin Ladybird", *Current Biology*, 28(20), 3296–3302, Copyright (2018), with permission from Elsevier.

Table S3. The metrics for measuring similarity between targets and generated morphs. A lower metric value means the morph is more similar to the target except SSIM. The values in bold are the best values for measuring similarity. MSE: mean squared error; CP: color proportion; SSIM: structural similarity index; VGG16PL: VGG16 perceptual loss; LPIPS: learned perceptual image patch similarity; VGG: VGG network; ALEX: AlexNet; SE: Squeeze–and–excitation network.

Morphs	Metrics	Targets			
		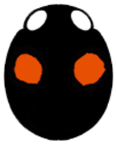	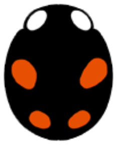	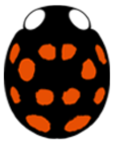	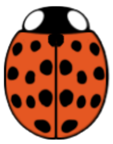
		conspicua	spectabilis	axyridis	succinea
	MSE ↓	**17.3242**	20.4783	28.9774	40.8210
	CP ↓	**0.2931**	0.3289	0.4202	20.3119
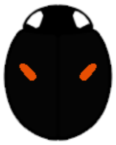	SSIM ↑	**0.5356**	0.4840	0.4117	0.3059
	VGG16PL ↓	**3196.0**	6166.0	11569.5	21204.2
	LPIPS:VGG ↓	**0.1718**	0.2459	0.3840	0.5168
morph-1	LPIPS:ALEX ↓	**0.1390**	0.1762	0.2981	0.4296
	LPIPS:SE ↓	**0.0737**	0.1570	0.2802	0.4042
	MSE ↓	**20.2113**	22.4684	31.6384	43.7115
	CP ↓	0.2721	0.2572	**0.2510**	1.8527
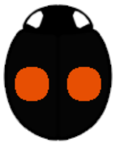	SSIM ↑	**0.5258**	0.4792	0.4006	0.3253
	VGG16PL ↓	**2783.8**	3551.8	8773.1	18108.6
	LPIPS:VGG ↓	**0.2519**	0.2892	0.4062	0.4856
morph-2	LPIPS:ALEX ↓	**0.2049**	0.2311	0.3179	0.4451
	LPIPS:SE ↓	**0.1335**	0.1772	0.2680	0.3608
	MSE ↓	**18.2089**	19.6686	28.5993	41.0735
	CP ↓	**0.2783**	0.3066	0.3807	15.0784
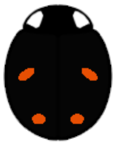	SSIM ↑	**0.5312**	0.4851	0.4133	0.3093
	VGG16PL ↓	**2894.8**	4798.3	10039.2	19797.5
	LPIPS:VGG ↓	0.2565	**0.1885**	0.3386	0.4948
morph-3	LPIPS:ALEX ↓	0.2306	**0.1441**	0.2613	0.4067
	LPIPS:SE ↓	0.1439	**0.0957**	0.2091	0.3454
	MSE ↓	**18.8651**	21.6270	29.7048	41.0292
	CP ↓	**0.2783**	0.3066	0.3807	15.0784
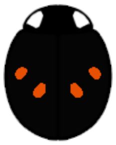	SSIM ↑	**0.5311**	0.4714	0.4034	0.3108
	VGG16PL ↓	**2784.6**	4871.5	10097.1	19894.1
	LPIPS:VGG ↓	**0.2373**	0.2737	0.3594	0.4969
morph-4	LPIPS:ALEX ↓	0.2365	**0.2131**	0.2432	0.3637
	LPIPS:SE ↓	**0.1412**	0.1742	0.2472	0.3640

Table S4. The metrics for measuring similarity between targets and generated morphs. See also Table S3 for details.

Morphs	Metrics	Targets			
		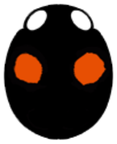 conspicua	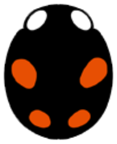 spectabilis	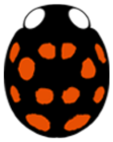 axyridis	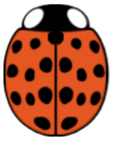 succinea
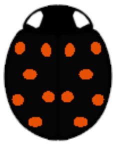 morph-5	MSE ↓	**21.1931**	22.6515	26.4112	43.0208
	CP ↓	**0.2509**	0.2529	0.2729	4.0632
	SSIM ↑	**0.4939**	0.4611	0.4413	0.2864
	VGG16PL ↓	9786.8	7374.6	**7035.6**	15944.1
	LPIPS:VGG ↓	0.3898	0.3464	**0.2334**	0.4611
	LPIPS:ALEX ↓	0.3173	0.2985	**0.1343**	0.2682
	LPIPS:SE ↓	0.2489	0.2071	**0.1168**	0.2501
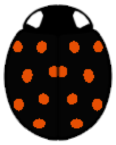 morph-6	MSE ↓	**21.2963**	22.8225	28.3330	42.6418
	CP ↓	**0.2507**	0.2537	0.2754	4.2744
	SSIM ↑	**0.4945**	0.4568	0.4197	0.2920
	VGG16PL ↓	10747.3	8366.4	**7449.8**	15995.7
	LPIPS:VGG ↓	0.4013	0.3600	**0.2791**	0.4476
	LPIPS:ALEX ↓	0.3465	0.3287	**0.1745**	0.2720
	LPIPS:SE ↓	0.2569	0.2207	**0.1371**	0.2494
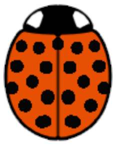 morph-7	MSE ↓	**34.4192**	35.5411	39.8690	50.4246
	CP ↓	8.8216	5.5762	2.8916	**0.2595**
	SSIM ↑	0.4378	0.4034	0.3730	**0.4499**
	VGG16PL ↓	28834.9	25995.5	22110.4	**14112.9**
	LPIPS:VGG ↓	0.5208	0.5003	0.4293	**0.1870**
	LPIPS:ALEX ↓	0.4556	0.4448	0.2596	**0.1213**
	LPIPS:SE ↓	0.3616	0.3130	0.2035	**0.0834**
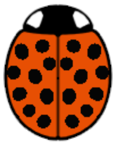 morph-8	MSE ↓	**34.3629**	34.5402	40.9031	50.3184
	CP ↓	5.8711	3.8163	2.0678	**0.2521**
	SSIM ↑	0.4319	0.4102	0.3680	**0.4460**
	VGG16PL ↓	29370.5	26509.5	22539.9	**14509.3**
	LPIPS:VGG ↓	0.5267	0.5013	0.4355	**0.1930**
	LPIPS:ALEX ↓	0.4574	0.4438	0.2579	**0.1393**
	LPIPS:SE ↓	0.3580	0.3079	0.2001	**0.0912**

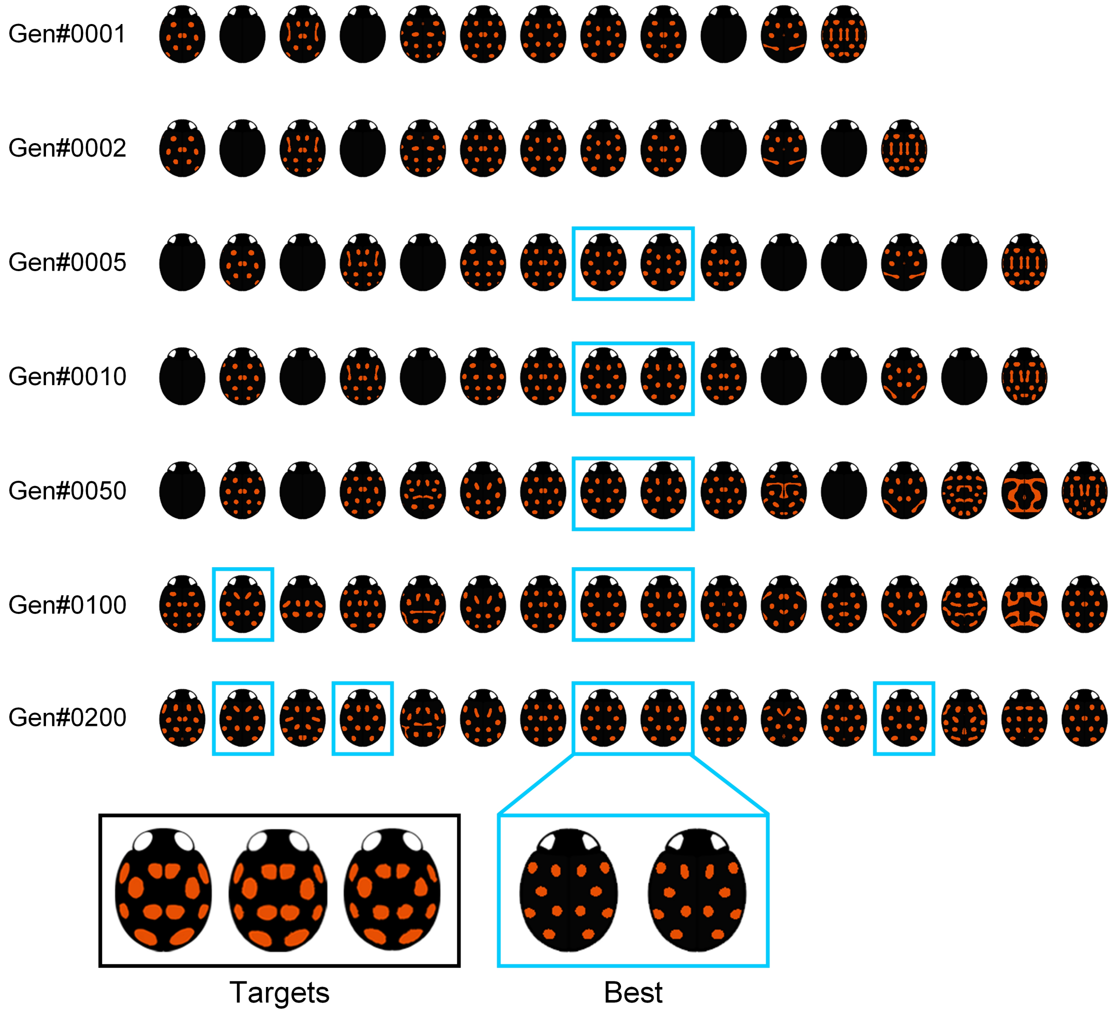

Figure S16. The generations of the axyridis subtype of *H. axyridis* in the evolutionary search. The maximum population size is 16, and the number of morphs in a generation is less than the maximum population size 16 means the parameter sets of the omitted morphs cause critical numerical errors resulting in invalid wing color patterns.

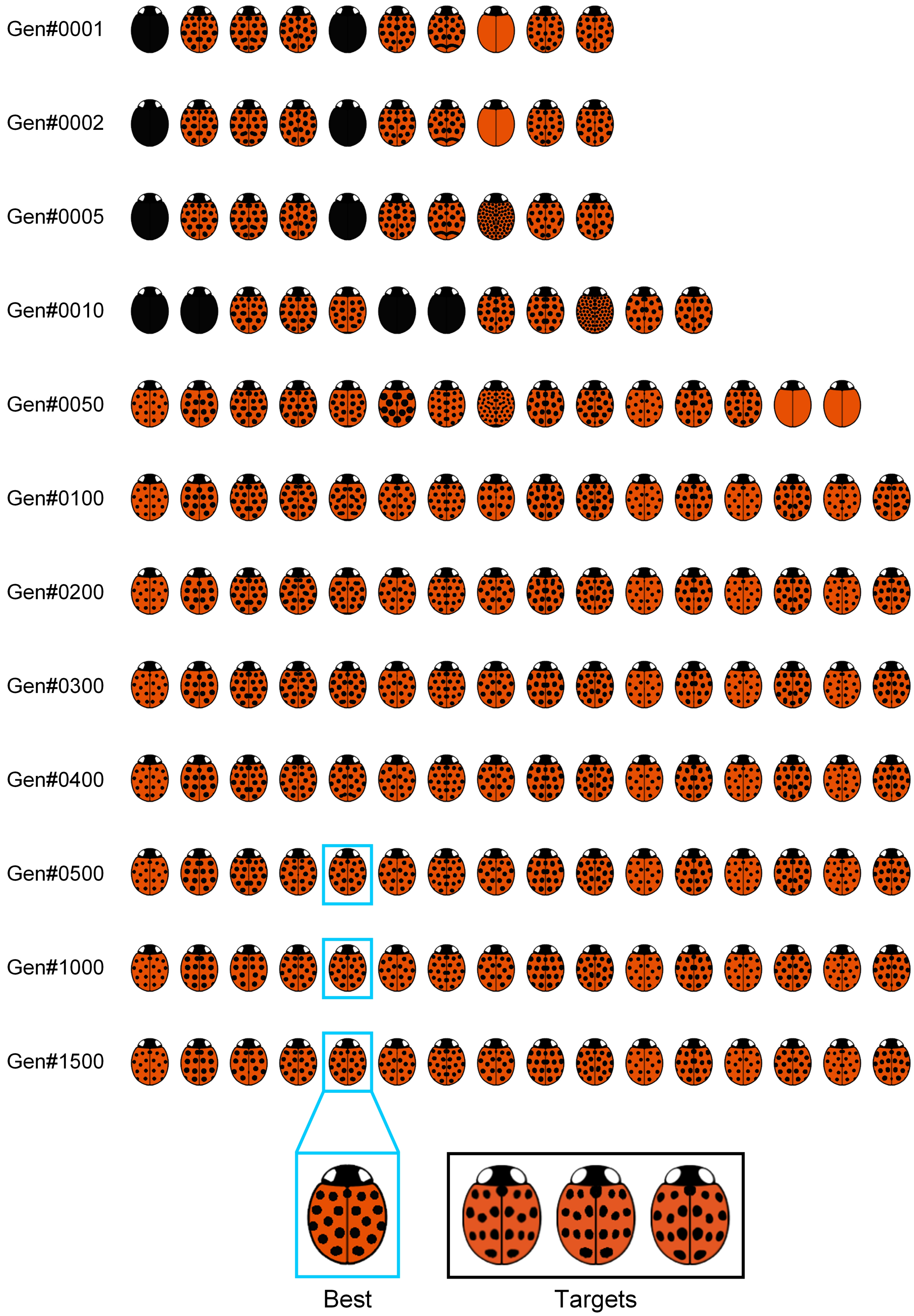

Figure S17. The generations of the succinea subtype of *H. axyridis* in the evolutionary search. The maximum population size is 16, and the number of morphs in a generation is less than the maximum population size 16 means the parameter sets of the omitted morphs cause critical numerical errors resulting in invalid wing color patterns.

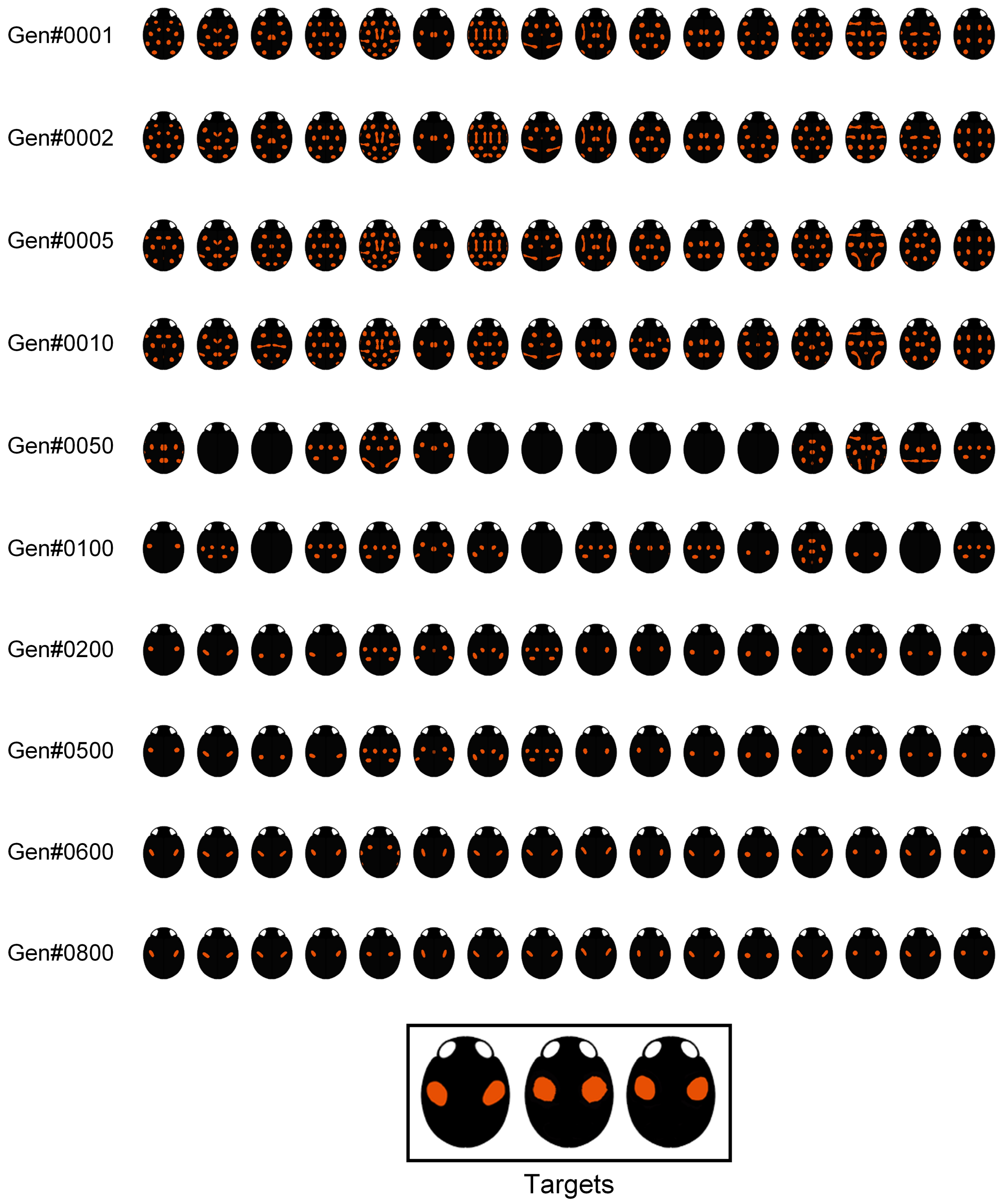

Figure S18. The generations of the conspicua subtype of *H. axyridis* in the evolutionary search. The maximum population size is 16.

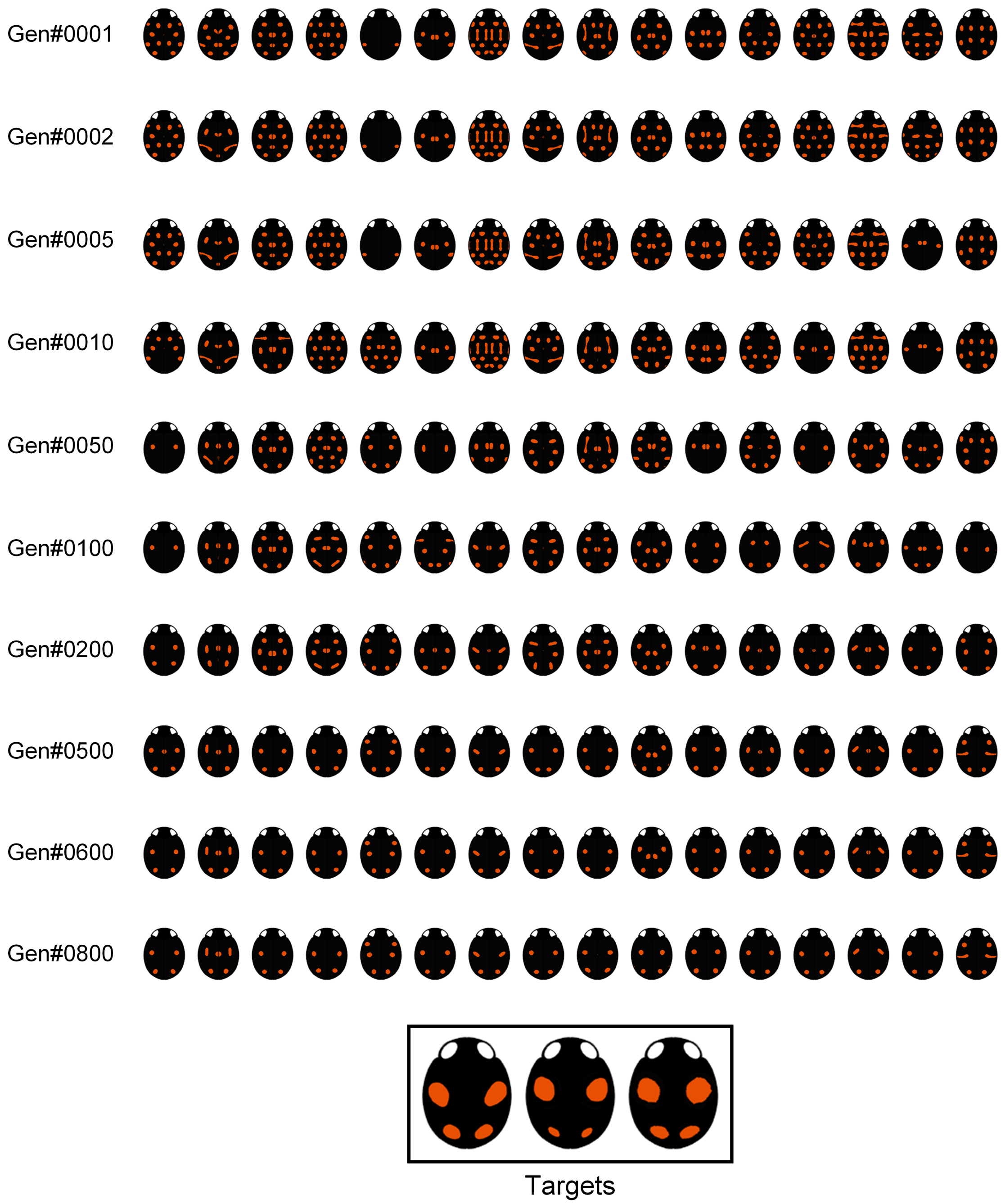

Figure S19. The generations of the spectabilis subtype of *H. axyridis* in the evolutionary search. The maximum population size is 16.

4.4 Results of a case study: reproducing real images

We also performed an evolutionary search for spectabilis subtype of *H. axyridis*, where the targets are noisy photos of spectabilis with asymmetric patterns captured in our laboratory (Figure S20). We use the Liaw model and the fitness score defined by Eq. (6), as in the section 4.3. Figure S21 shows the search results of finding mathematical models to reproduce noisy photos. In some generations, many morphs are missing because parameter sets generated during the evolution caused critical numerical errors. After 300 generations, we can observe morphs with four red spots similar to the target photos (Figure S21). These results suggest that we can discover mathematical models that can reproduce real images captured in the laboratory using LPF. However, note that noisy and asymmetric patterns of real photos might cause numerical errors, which can result in omitted morphs in the evolution process.

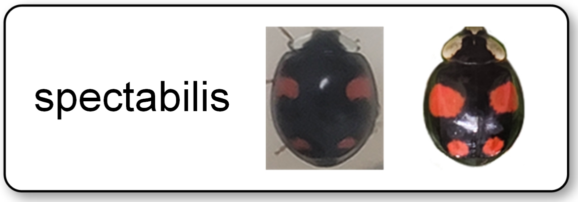

Figure S20. Noisy photos of *H. axyridis* spectabilis with asymmetric patterns.

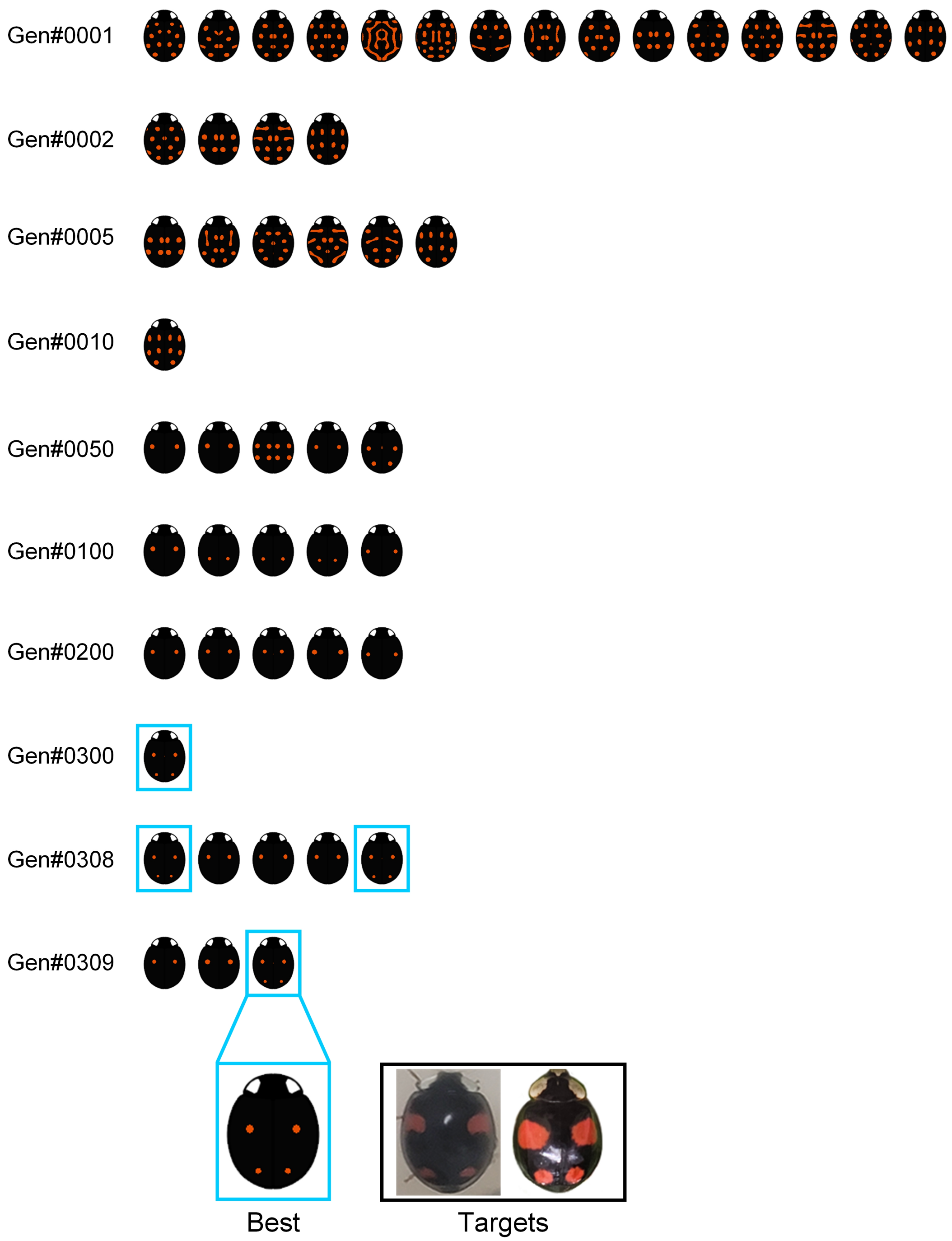

Figure S21. The generations of the spectabilis subtype of *H. axyridis* in the evolutionary search, where the targets are noisy photos of *H. axyridis* spectabilis with asymmetric patterns captured in the laboratory. The maximum population size is 16.

5 Diploid model

Genetic crossing experiments substantiated that the prevalence of wing color patterns in *H. axyridis* originates from the variety of numerous alleles segregating at a single autosomal locus [Tan and Li, 1934, Komai, 1956]. Tan proposed 'mosaic dominance' phenomena, which suggests that the color pattern of a heterozygote is a combination of color patterns of the two alleles in *H. axyridis* [Tan, 1946, Ando and Niimi, 2019] Recently, Ando *et al.* and Gautier *et al.* discovered that repeated inversion within a *pannier* intron is the source of color pattern polymorphism of *H. axyridis* [Ando et al., 2018, Gautier et al., 2018].

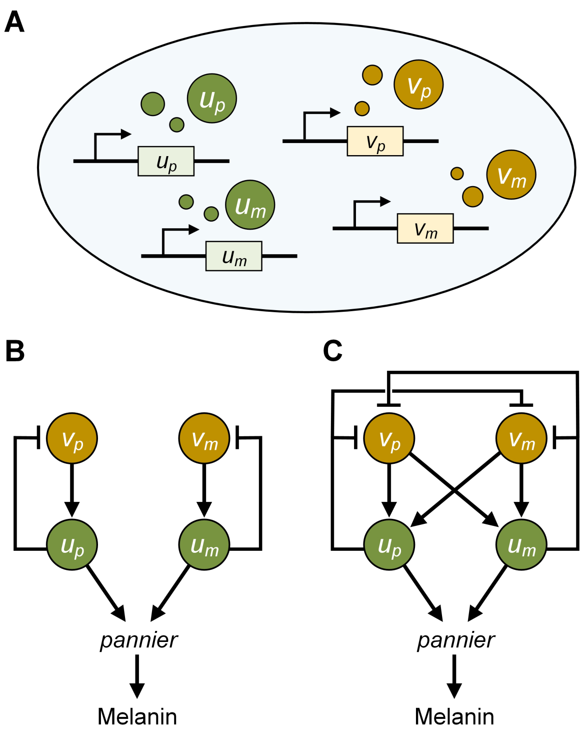

Figure S22. Diploid models of *H. axyridis* in LPF. **(A)** The diploid models contain paternal and maternal alleles for each genetic locus. Squares represent genes and circles represent proteins in the cell. **(B)** A two-component diploid model without crosstalks. **(C)** A two-component diploid model with crosstalks.

To reflect the genetic features of *H. axyridis* in inheritance, we have developed diploid models (Figure S22). We assume that the expression of *pannier* is regulated by hypothetical morphogens, which are expressed from paternal and maternal alleles (Figure S22A, u_p and u_m). We have also considered the presence of crosstalks between u and v in the diploid models (Figure S22B and C). In a two-component system, a PDE model without crosstalks can be defined by Eq. (13).

$$\begin{aligned}
\frac{\partial u_p}{\partial t} &= D_{u_p}\nabla^2 u_p + f_p(u_p, v_p),\\
\frac{\partial v_p}{\partial t} &= D_{v_p}\nabla^2 v_p + g_p(u_p, v_p),\\
\frac{\partial u_m}{\partial t} &= D_{u_m}\nabla^2 u_m + f_m(u_m, v_m),\\
\frac{\partial v_m}{\partial t} &= D_{v_m}\nabla^2 v_m + g_m(u_m, v_m),
\end{aligned} \tag{13}$$

where m and p represent paternal and maternal origins. The total u and v of the progeny model can be defined by a linear combination of paternal and maternal states (Eq. (14)).

$$\begin{aligned}
u &= \alpha u_p + \beta u_m,\\
v &= \alpha v_p + \beta v_m,
\end{aligned} \tag{14}$$

where α and β are the coefficients of the linear combination. On the other hand, the crosstalk model of a two-component system can be defined by Eq. (15).

$$\begin{aligned}
\frac{\partial u}{\partial t} &= D_u\nabla^2 u + \alpha f_p(u, v) + \beta f_m(u, v),\\
\frac{\partial v}{\partial t} &= D_v\nabla^2 v + \alpha g_p(u, v) + \beta g_m(u, v),
\end{aligned} \tag{15}$$

where the reactions are modeled by a linear combination of paternal and maternal reactions. The models of Eqs. (13), (14), and (15) are implemented as `TwoComponentDiploidModel` and `TwoComponentCrosstalkDiploidModel` in LPF.

5.1 Numerical simulation

We performed simulation experiments, where succinea and conspicua models of *H. axyridis* are crossed (Figure S23A). Code 18 shows an example of how to obtain a numerical solution using the diploid models of LPF. In the crossing experiments, the diploid model without crosstalks is able to reproduce the mosaic dominance phenomena (Figure S23B). However, the color patterns of the crosstalk model are far from the patterns of mosaic dominance (Figure S23C). Since the regulators of *pannier* are not fully understood, these diploid models of LPF are expected to be useful for testing various hypotheses about the underlying mechanisms of ladybird polymorphism.

Code 18. An example of obtaining a numerical solution of diploid models.

```python
import os
import os.path as osp
from os.path import join as pjoin
import time
from datetime import datetime

import numpy as np

from lpf.initializers import LiawInitializer
from lpf.models import LiawModel
from lpf.models import TwoComponentDiploidModel
from lpf.models import TwoComponentCrosstalkDiploidModel
from lpf.solvers import EulerSolver
from lpf.visualization import merge_single_timeseries

if __name__ == "__main__":

    device = "cpu"
    dx = 0.1
    dt = 0.01
    width = 128
    height = 128
    thr = 0.5
    n_iters = 500000
    shape = (height, width)

    # Create the output directory.
    LPF_REPO_HOME = ... # Specify the directory path of LPF repository.
    str_now = datetime.now().strftime('%Y%m%d-%H%M%S')
    dpath_output = pjoin(LPF_REPO_HOME,
                         "experiments",
                         "output",
                         "diploid_%s" % (str_now))
    os.makedirs(osp.abspath(dpath_output), exist_ok=True)

    # Create a paternal model.
    model_dict = [
        {"u0": 1.5635296830073362,
         "v0": 1.6325853074866885,
         "Du": 0.0004980801662982812,
         "Dv": 0.07500000000000001,
         "ru": 0.17999999999999997,
         "rv": 0.0979337206999831,
         "k": 0.20000000000000004,
         "su": 0.0008394576268270522,
```

```
         "sv": 0.025000000000000005,
         "mu": 0.07999999999999999,
         "init_pts_0": ["33", "60"],
         "init_pts_1": ["46", "3"],
         "init_pts_2": ["53", "50"],
         "init_pts_3": ["39", "70"],
         "init_pts_4": ["40", "90"],
         "init_pts_5": ["64", "4"],
         "init_pts_6": ["60", "117"],
         "init_pts_7": ["66", "7"],
         "init_pts_8": ["50", "70"],
         "init_pts_9": ["50", "90"],
         "init_pts_10": ["58", "48"],
         "init_pts_11": ["60", "30"],
         "init_pts_12": ["63", "47"],
         "init_pts_13": ["87", "30"],
         "init_pts_14": ["77", "4"],
         "init_pts_15": ["87", "29"],
         "init_pts_16": ["72", "111"],
         "init_pts_17": ["57", "61"],
         "init_pts_18": ["110", "42"],
         "init_pts_19": ["78", "59"]}
    ]

    # Create an initializer.
    initializer = LiawInitializer()
    initializer.update(model_dict)

    params = LiawModel.parse_params(model_dict)
    pa_model = LiawModel(
        initializer=initializer,
        params=params,
        width=width,
        height=height,
        dx=dx,
        device=device
    )

    # Create a maternal model.
    model_dict = [
        {"u0": 4.125239430041862,
         "v0": 18.18479114252238,
         "Du": 0.0011041379940844168,
         "Dv": 0.14596920545639724,
         "ru": 0.08897465243231621,
         "rv": 0.11404666216196024,
         "k": 0.5720189763213703,
         "su": 0.0007382506069868803,
         "sv": 0.021257567063086704,
```

```
        "mu": 0.028948565299659442,
        "init_pts_0": ["89", "93"],
        "init_pts_1": ["25", "65"],
        "init_pts_2": ["77", "52"],
        "init_pts_3": ["62", "96"],
        "init_pts_4": ["12", "88"],
        "init_pts_5": ["27", "67"],
        "init_pts_6": ["26", "28"],
        "init_pts_7": ["44", "81"],
        "init_pts_8": ["86", "44"],
        "init_pts_9": ["80", "69"],
        "init_pts_10": ["42", "72"],
        "init_pts_11": ["90", "50"],
        "init_pts_12": ["61", "30"],
        "init_pts_13": ["63", "71"],
        "init_pts_14": ["16", "23"],
        "init_pts_15": ["91", "88"],
        "init_pts_16": ["35", "33"],
        "init_pts_17": ["81", "2"],
        "init_pts_18": ["70", "89"],
        "init_pts_19": ["83", "75"]}
    ]

    # Create an initializer.
    initializer = LiawInitializer()
    initializer.update(model_dict)

    params = LiawModel.parse_params(model_dict)
    ma_model = LiawModel(
        initializer=initializer,
        params=params,
        width=width,
        height=height,
        dx=dx,
        device=device
    )

    # Create a diploid model with paternal and maternal models.
    model = TwoComponentDiploidModel(
        paternal_model=pa_model,
        maternal_model=ma_model,
        alpha=0.5,
        beta=0.5,
        device=device
    )

    # Perform a numerical simulation.
    solver = EulerSolver()

```

```python
    t_beg = time.time()
    solver.solve(
        model=model,
        dt=dt,
        n_iters=n_iters,
        period_output=5000,
        dpath_model=dpath_output,
        dpath_ladybird=dpath_output,
        dpath_pattern=dpath_output,
        verbose=1
    )

    t_end = time.time()
    print("Elapsed time: %f sec." % (t_end - t_beg))

    # Visualize the results.
    dpath_images = pjoin(dpath_output, "model_1")

    img_patterns = merge_single_timeseries(dpath_input=dpath_images,
                                           n_cols=10,
                                           infile_header="pattern",
                                           ratio_resize=0.5,
                                           text_format="n = ",
                                           font_size=10,
                                           text_margin_ratio=0.1)

    img_patterns.save(pjoin(dpath_output, "output_pattern.png"))

    img_ladybirds = merge_single_timeseries(dpath_input=dpath_images,
                                            n_cols=10,
                                            infile_header="ladybird",
                                            ratio_resize=0.5,
                                            text_format="n = ",
                                            font_size=10,
                                            text_margin_ratio=0.1)

    img_ladybirds.save(pjoin(dpath_output, "output_ladybird.png"))
```

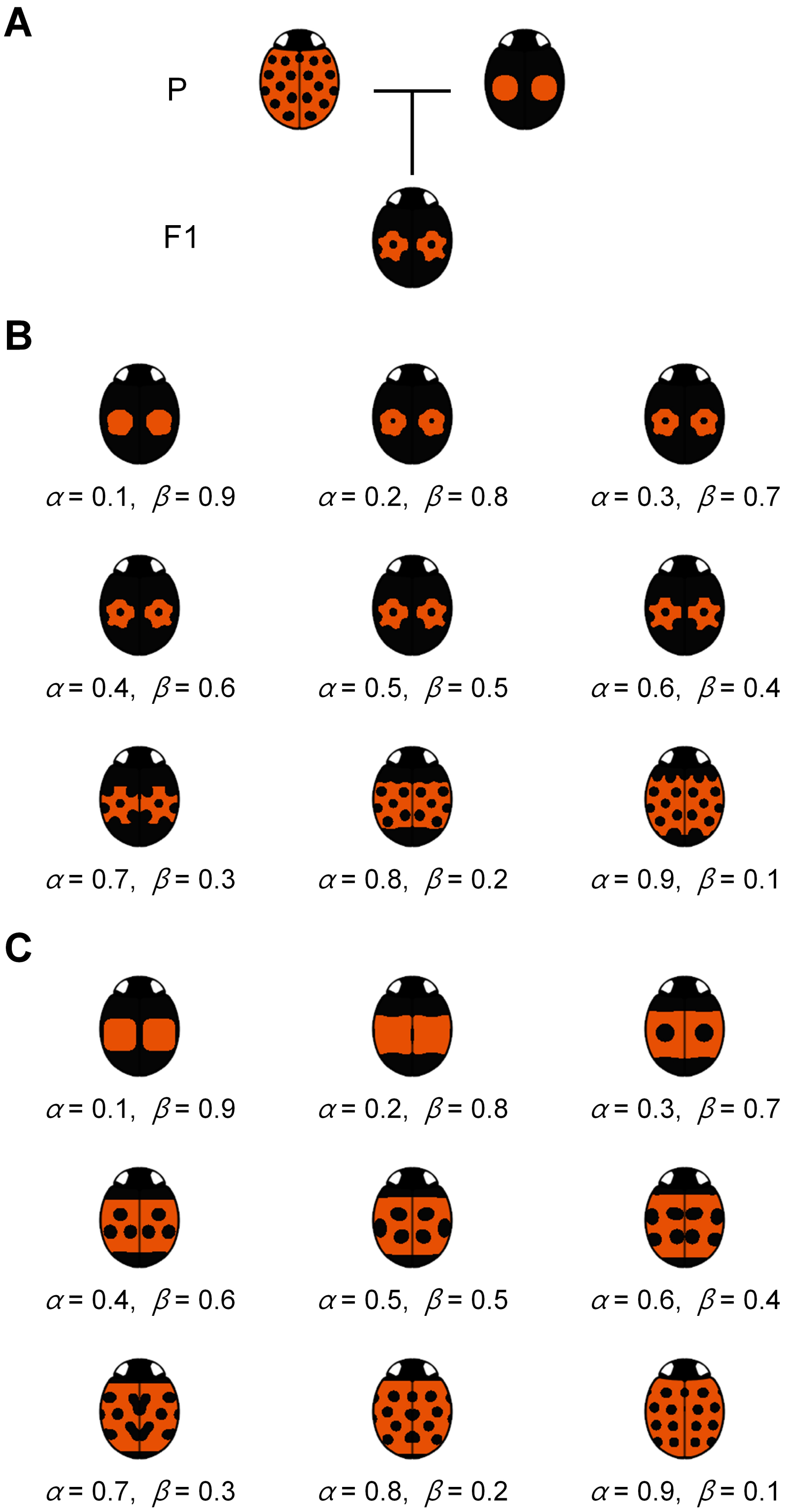

Figure S23. The numerical simulation results of `TwoComponentDiploidModel` for *H. axyridis*. The paternal and maternal models are the Liaw model. F1 denotes the progeny of the crossed organisms. **(A)** The succinea (male) and conspicua (female) models are crossed. The progenies obtained from different α and β combinations for **(B)** `TwoComponentModel` and **(C)** `TwoComponentCrosstalkModel`.

5.2 Population evolution

We performed crossing experiments for population evolution, where succinea and conspicua subtypes of Figure S23A are crossed in the initial population. In this experiment, we have adopted a concept of crossover in genetics. In the early stages of meiosis, a chromosome duplicated into two identical sister chromatids is paired with a homologous chromosome from the other parent. This pair forms a structure called a tetrad. DNA segments of paternal and maternal chromosomes in the tetrad can be exchanged, which is a process known as crossover. We assumed the crossover between paternal and maternal alleles occurs according to a user-defined probability. For example, if the total 32 haploid daughter cells (i.e., gametes or haploid models) are generated from a parent diploid model, 16 daughter models can have alleles produced by the crossover when the probability of crossover is 0.5. For simplicity, we implemented an *in silico* crossover that could randomly occur on any single parameter, initial state, and initializing position between the haploid models of a diploid model (Figure S24). A progeny model is created by selecting paternal and maternal haploid models (i.e., gametes) and creating a diploid model based on the haploid models. To obtain the next generation population, we repeated crossing experiments. For example, if we repeat the crossing experiment four times to build a population of 32 individuals, we should get eight offspring individuals from a single crossing experiment.

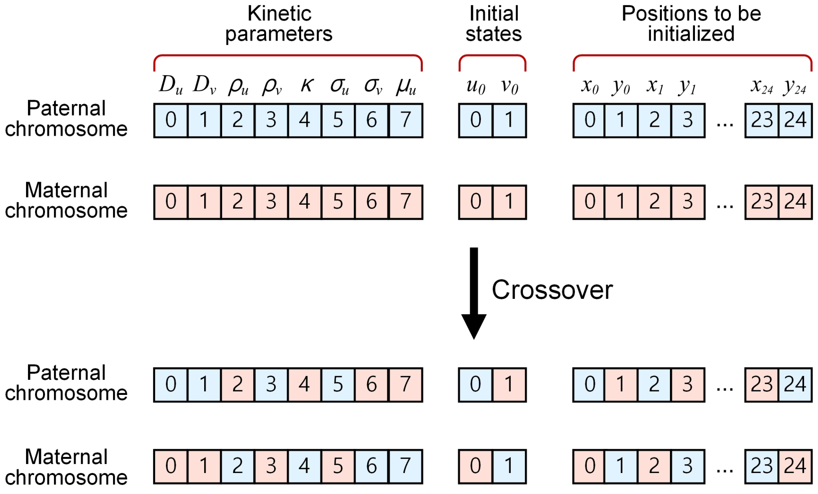

Figure S24. An example of *in silico* crossover of a diploid model, where the paternal and maternal haploid models are Liaw model. It is assumed that the exchange of values between paternal and maternal models randomly occurs.

Code 19 is an example of how to perform the population evolution based on `LiawModel` and `TwoComponentDiploidModel` in LPF. Figure S25 and Figure S26 show the results of the population evolution from the two duplicate experiments. In the early generations

of both experiments, we can see the morphs of mosaic dominance (Figure S23A). Interestingly, the individuals that make up the final populations are entirely different due to random selection and random crossover, although the experiments were conducted under identical conditions. Another interesting observation is that a few morphs dominate the population after generations, suggesting a kind of genetic drift.

Code 19. An example of evolving a diploid population by crossing.

```
import os
import os.path as osp
from os.path import join as pjoin
from datetime import datetime

import numpy as np

from lpf.initializers import LiawInitializer
from lpf.models import LiawModel
from lpf.models import TwoComponentDiploidModel
from lpf.solvers import EulerSolver
from lpf.reproducers import RandomTwoComponentDiploidReproducer


if __name__ == "__main__":

    # Define computing device.
    device = "cuda:0"

    # Define spatiotemporal parameters.
    dx = 0.1
    width = 128
    height = 128
    thr_color = 0.5
    dt = 0.01
    n_iters = 500000

    # Define hyper-parameters for population evolution.
    n_generations = 1000
    pop_size = 32  # Size of population (the number of organisms)
    n_cross = 4  # Number of crossing experiments
    n_gametes = 32  # Number of gametes (the number of daughter cells)
    prob_crossover = 0.3  # Probability that crossover occurs
    alpha = 0.5
    beta = 0.5

    # Create the output directory.
    LPF_REPO_HOME = ... # Specify the directory path of LPF repository.
    str_now = datetime.now().strftime('%Y%m%d-%H%M%S')
```

```
    dpath_output = pjoin(LPF_REPO_HOME,
                         "search",
                         "output",
                         "evopop_%s"%(str_now))
    dpath_output = osp.abspath(dpath_output)
    os.makedirs(dpath_output, exist_ok=True)

    # Create a paternal haploid model.
    model_dict = [
        {"u0": 1.5635296830073362,
         "v0": 1.6325853074866885,
         "Du": 0.0004980801662982812,
         "Dv": 0.07500000000000001,
         "ru": 0.17999999999999997,
         "rv": 0.0979337206999831,
         "k": 0.20000000000000004,
         "su": 0.0008394576268270522,
         "sv": 0.025000000000000005,
         "mu": 0.07999999999999999,
         "init_pts_0": ["33", "60"],
         "init_pts_1": ["46", "3"],
         "init_pts_2": ["53", "50"],
         "init_pts_3": ["39", "70"],
         "init_pts_4": ["40", "90"],
         "init_pts_5": ["64", "4"],
         "init_pts_6": ["60", "117"],
         "init_pts_7": ["66", "7"],
         "init_pts_8": ["50", "70"],
         "init_pts_9": ["50", "90"],
         "init_pts_10": ["58", "48"],
         "init_pts_11": ["60", "30"],
         "init_pts_12": ["63", "47"],
         "init_pts_13": ["87", "30"],
         "init_pts_14": ["77", "4"],
         "init_pts_15": ["87", "29"],
         "init_pts_16": ["72", "111"],
         "init_pts_17": ["57", "61"],
         "init_pts_18": ["110", "42"],
         "init_pts_19": ["78", "59"]}
    ]

    # Create an initializer.
    initializer = LiawInitializer()
    initializer.update(model_dict)

    params = LiawModel.parse_params(model_dict)
    pa_model = LiawModel(
        initializer=initializer,
```

```
        params=params,
        width=width,
        height=height,
        dx=dx,
        device=device
    )

    # Create a maternal haploid model.
    model_dict = [
        {"u0": 4.125239430041862,
         "v0": 18.18479114252238,
         "Du": 0.0011041379940844168,
         "Dv": 0.14596920545639724,
         "ru": 0.08897465243231621,
         "rv": 0.11404666216196024,
         "k": 0.5720189763213703,
         "su": 0.0007382506069868803,
         "sv": 0.021257567063086704,
         "mu": 0.028948565299659442,
         "init_pts_0": ["89", "93"],
         "init_pts_1": ["25", "65"],
         "init_pts_2": ["77", "52"],
         "init_pts_3": ["62", "96"],
         "init_pts_4": ["12", "88"],
         "init_pts_5": ["27", "67"],
         "init_pts_6": ["26", "28"],
         "init_pts_7": ["44", "81"],
         "init_pts_8": ["86", "44"],
         "init_pts_9": ["80", "69"],
         "init_pts_10": ["42", "72"],
         "init_pts_11": ["90", "50"],
         "init_pts_12": ["61", "30"],
         "init_pts_13": ["63", "71"],
         "init_pts_14": ["16", "23"],
         "init_pts_15": ["91", "88"],
         "init_pts_16": ["35", "33"],
         "init_pts_17": ["81", "2"],
         "init_pts_18": ["70", "89"],
         "init_pts_19": ["83", "75"]}
    ]

    # Create an initializer.
    initializer = LiawInitializer()
    initializer.update(model_dict)

    params = LiawModel.parse_params(model_dict)
    ma_model = LiawModel(
        initializer=initializer,
        params=params,
```

```
        width=width,
        height=height,
        dx=dx,
        device=device
    )

    # Create a population.
    init_pop = []

    for i in range(pop_size):
        model = TwoComponentDiploidModel(
            paternal_model=pa_model,
            maternal_model=ma_model,
            alpha=alpha,
            beta=beta,
            device=device
        )
        init_pop.append(model)
    # end of for

     # Create a solver.
    solver = EulerSolver(
        dt=dt,
        n_iters=n_iters,
        period_output=n_iters,
        verbose=0
    )

    # Create a reproducer.
    reproducer = RandomTwoComponentDiploidReproducer(
        population=init_pop,
        solver=solver,
        pop_size=pop_size,
        n_cross=n_cross,
        n_gametes=n_gametes,
        prob_crossover=prob_crossover,
        alpha=alpha,
        beta=beta,
        diploid_model_class=TwoComponentDiploidModel,
        haploid_model_class=LiawModel,
        haploid_initializer_class=LiawInitializer,
        dpath_output=dpath_output,
        device=device,
        verbose=1
    )

    # Start the evolution of population.
    reproducer.evolve(n_generations=n_generations)

```

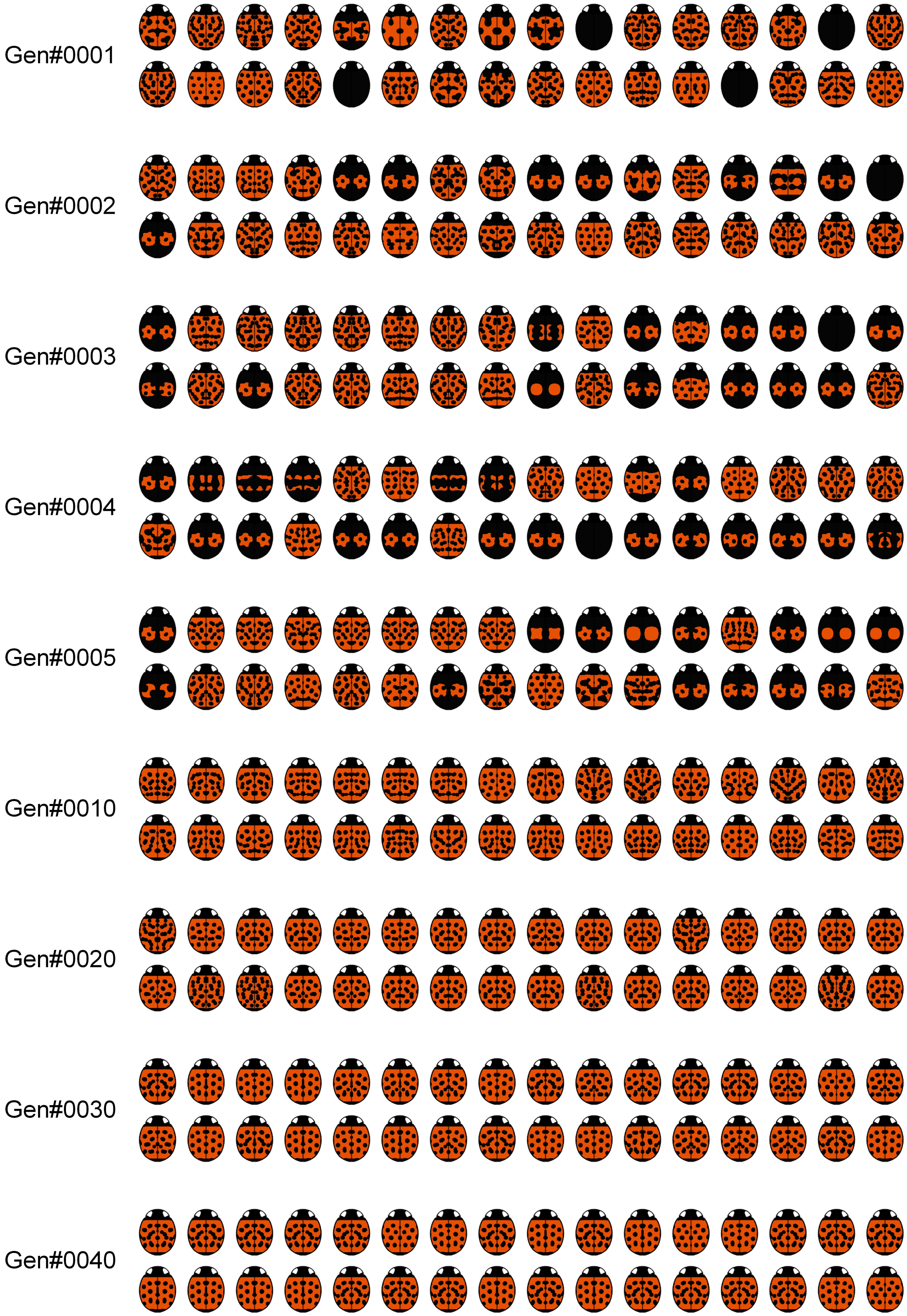

Figure S25. The generations of a population evolution, where the initial population consists of the progenies of *H. axyridis* succinea and spectabilis subtypes. The maximum population size is 32.

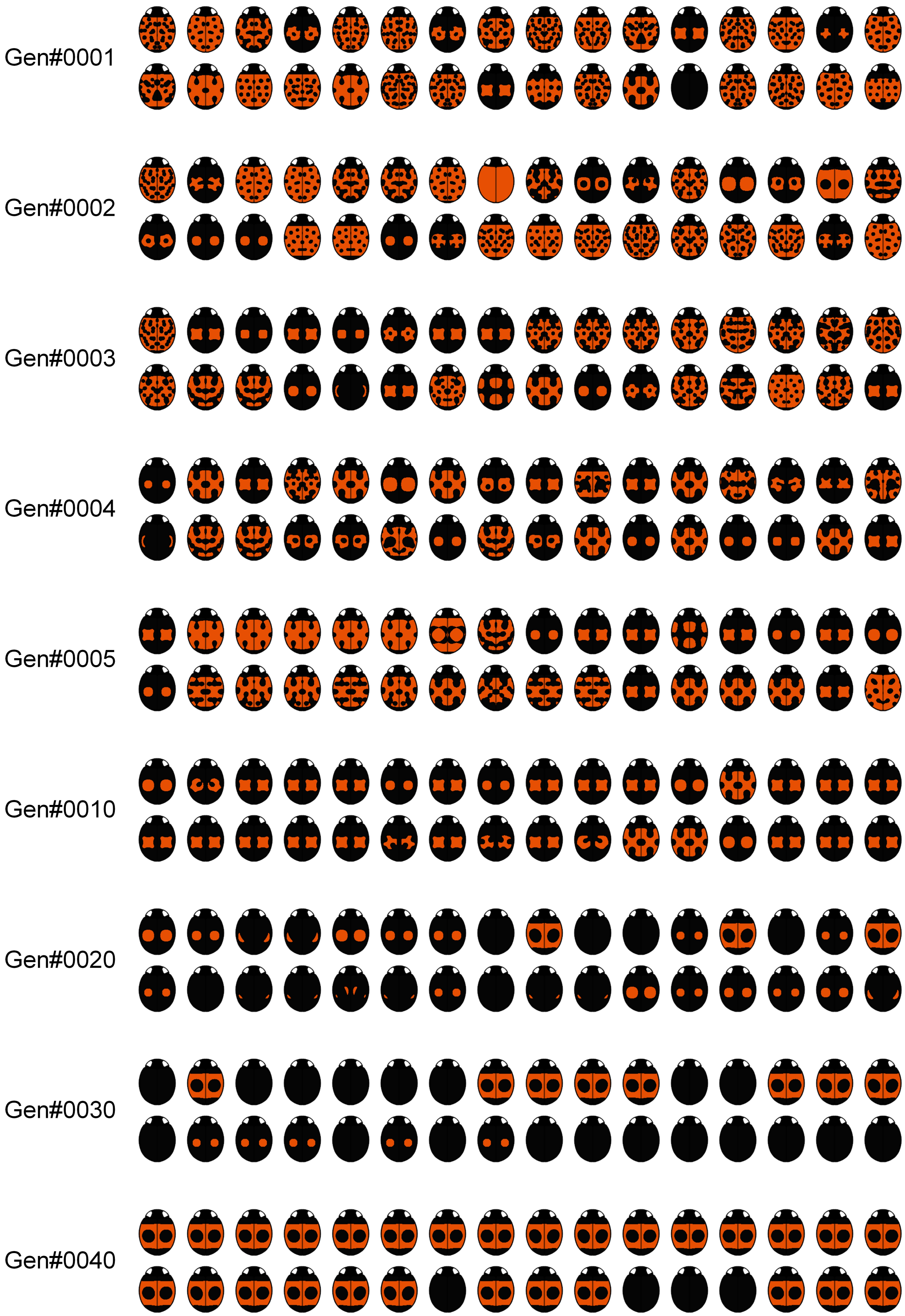

Figure S26. The generations of a population evolution, where the initial population consists of the progenies of *H. axyridis* succinea and spectabilis subtypes. The maximum population size is 32.

References

T. Ando and T. Niimi. Development and evolution of color patterns in ladybird beetles: A case study in harmonia axyridis. *Development, Growth & Differentiation*, 61(1): 73–84, 2019. doi: https://doi.org/10.1111/dgd.12592.

T. Ando, T. Matsuda, K. Goto, K. Hara, A. Ito, J. Hirata, J. Yatomi, R. Kajitani, M. Okuno, K. Yamaguchi, et al. Repeated inversions within a *Pannier* intron drive diversification of intraspecific colour patterns of ladybird beetles. *Nature Communications*, 9(1):1–13, 2018.

F. Biscani and D. Izzo. A parallel global multiobjective framework for optimization: pagmo. *Journal of Open Source Software*, 5(53):2338, 2020. doi: 10.21105/joss.02338.

G. Bradski. The OpenCV Library. *Dr. Dobb's Journal of Software Tools*, 2000.

M. Gautier, J. Yamaguchi, J. Foucaud, A. Loiseau, A. Ausset, B. Facon, B. Gschloessl, J. Lagnel, E. Loire, H. Parrinello, et al. The genomic basis of color pattern polymorphism in the harlequin ladybird. *Current Biology*, 28(20):3296–3302, 2018.

A. Gierer and H. Meinhardt. A theory of biological pattern formation. *Kybernetik*, 12: 30–39, 1972.

P. Gray and S. K. Scott. Autocatalytic reactions in the isothermal, continuous stirred tank reactor: Oscillations and instabilities in the system a+ 2b$\rightarrow$ 3b; b$\rightarrow$ c. *Chemical Engineering Science*, 39(6):1087–1097, 1984.

T. Komai. Genetics of ladybeetles. *Advances in Genetics*, 8:155–188, 1956.

C. Ledig, L. Theis, F. Huszár, J. Caballero, A. Cunningham, A. Acosta, A. Aitken, A. Tejani, J. Totz, Z. Wang, et al. Photo-realistic single image super-resolution using a generative adversarial network. In *Proceedings of the IEEE Conference on Computer Vision and Pattern Recognition*, pages 4681–4690, Honolulu, HI, USA, 2017.

S. S. Liaw, C. C. Yang, R. T. Liu, and J. T. Hong. Turing model for the patterns of lady beetles. *Physical Review E*, 64:041909, Sep 2001. doi: 10.1103/PhysRevE.64.041909.

S. Ocklenburg and A. Mundorf. Symmetry and asymmetry in biological structures. *Proceedings of the National Academy of Sciences*, 119(28):e2204881119, 2022.

R. Okuta, Y. Unno, D. Nishino, S. Hido, and C. Loomis. Cupy: A numpy-compatible library for nvidia gpu calculations. In *Proceedings of Workshop on Machine Learning*

Systems in the 31st Annual Conference on Neural Information Processing Systems (NIPS), Long Beach, CA, USA, 2017.

J. Schnakenberg. Simple chemical reaction systems with limit cycle behaviour. *Journal of Theoretical Biology*, 81(3):389–400, 1979.

C. Tan. Mosaic dominance in the inheritance of color patterns in the lady-bird beetle, harmonia axyridis. *Genetics*, 31(2):195, 1946.

C.-C. Tan and J.-C. Li. Inheritance of the elytral color patterns of the lady-bird beetle, harmonia axyridis pallas. *The American Naturalist*, 68(716):252–265, 1934.

R. Y. Zhang, P. Isola, A. A. Efros, E. Shechtman, and O. Wang. The unreasonable effectiveness of deep features as a perceptual metric. In *Proceedings of the IEEE Conference on Computer Vision and Pattern Recognition (CVPR)*, pages 586–595, Salt Lake City, UT, USA, 2018.

Zulko. Moviepy. https://github.com/Zulko/moviepy, 2020.
